# Additive-free hyaluronic acid-based bioink for 3D bioprinting of bone marrow microenvironments

**DOI:** 10.1016/j.mtbio.2025.102512

**Published:** 2025-11-05

**Authors:** Toufik Naolou, Nadine Schadzek, Jonas Nolte, Susanna Spindler, Franziska Lötz, Lena Fraedrich, Gerald Dräger, Tomasz Jüngst, Jürgen Groll, Cornelia Lee-Thedieck

**Affiliations:** a Institute of Cell Biology and Biophysics, Leibniz University Hannover, Herrenhäuser Str. 2, 30419, Hannover, Germany; b Institute of Organic Chemistry, Leibniz University Hannover, Schneiderberg 1B, 30167, Hannover, Germany; c Department for Functional Materials in Medicine and Dentistry, University of Würzburg, Pleicherwall 2, 97070, Würzburg, Germany; d Hannover Medical School, Institute of Neuroanatomy and Cell Biology, Carl-Neuberg-Straße 1, 30625 Hannover, Germany

**Keywords:** Hyaluronic acid, Extrusion-based bioprinting, Bioink, Bone marrow, Hematopoietic stem cells (HSCs), Mesenchymal stem/stromal cells (MSCs)

## Abstract

Bioprinting of soft tissues is an emerging technology with significant potential in regenerative medicine. It requires bioinks that mimic biochemical and physical properties of natural tissues and enable precise positioning of multiple cell types to allow their physiological interplay. Here, we describe the development of a bioink for bone marrow as an example of soft tissue. Bone marrow is the site of blood regeneration, driven by hematopoietic stem cells (HSCs) and relying on the orchestrated interplay of hematopoietic and stromal cells in a soft microenvironment. The bioink is based on hyaluronic acid (HA), dual-functionalized in a one-pot synthesis with alkyl side chains enhancing physical crosslinking via hydrophobic interactions and methacrylamide groups allowing covalent photo-crosslinking. Polymers are synthesized with HA of differing molecular weights, alkyl side chain lengths and modification degrees. Their gelling behavior, shear-thinning and self-healing properties deem them suitable for extrusion-based bioprinting. The ink allows two bioprinting approaches: cell encapsulation pre-printing and cell injection post-printing, both yielding excellent cell viability. The latter approach allows precise placement of hematopoietic and stromal cells in a single construct.

In summary, we present a bioink enabling bioprinting of modified HA without further additives, bioprinting of encapsulated cells and injection of cells into pre-printed structures. The material is based on a polymer naturally present in bone marrow, resembles the mechanical properties of bone marrow and is suitable for bioprinting of hematopoietic and stromal cells. Thus, this bioink is a promising platform for bioprinting biomimetic bone marrow or other soft tissue constructs for future fundamental and applied research.

## Highlights

• One-pot synthesis of dual-functionalized hyaluronic acid for bioprinting.
• Alkyl side chains enhance physical, methacrylate groups allow covalent crosslinking.
• Optimal material characteristics for extrusion-based printing without additives.
• Cell encapsulation and injection enabled, allowing precise positioning of multiple cell types.
• Bioink mimicking bone marrow matrix as platform for *in vitro* models or soft tissue engineering.

## Graphical abstract

**Figure:**
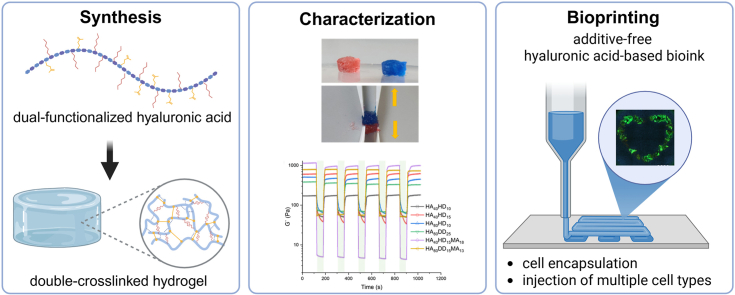

## 1 Introduction

Bioprinting has emerged as a valuable technology for fabricating complex, biologically functional constructs with precise spatial control [1]. Among the bioprinting technologies, extrusion-based bioprinting stands out for enabling the deposition of hydrogel-based bioinks laden with living cells, growth factors, or other bioactive molecules. This approach allows for the precise combination of hydrogels with distinct mechanical properties and/or cell types while maintaining full spatial control within the bioprinted scaffolds to mimic the hierarchical organization of the natural tissue [2].

Soft tissue engineering is emerging for clinical and pharmaceutical applications. Among these, the bone marrow is an interesting archetype of a soft tissue, due to its vital regenerative capacities including the hematopoietic, i.e. the blood building system of the body. In specialized stem cell niches in the bone marrow, hematopoietic stem cells (HSCs) are able to self-renew and differentiate into blood and immune cells. This is allowed by a dynamic interplay of diverse cell types within a specialized 3D microenvironment, homing these cells [[3], [4], [5]]. To gain a deeper understanding of the processes involved in the human hematopoietic system in both health and disease, artificial stem cell niches created *in vitro* serve as valuable tools in fundamental research. Moreover, such systems are promising for clinical or pharmaceutical applications, such as the production of cells for cellular therapies or *in vitro* drug testing, respectively [6]. However, the translational potential of bone marrow models is limited by the degree of biomimicry and natural cell behavior that can be achieved. Therefore, enhancing the similarity of the models to the *in vivo*-situation and thus raising their complexity to the required degree, while keeping them as simple as possible to allow robust application, could unlock the potential of *in vitro* bone marrow models, e.g., for disease modeling, personalized drug screening, or multiplication of HSCs for transplantation of patients suffering from hematological diseases such as leukemia [[6], [7], [8]].

For rebuilding HSC niches *in vitro,* the cellular composition, the extracellular matrix (ECM) components and the biophysical properties of the natural niche have to be considered. A variety of cell types, including osteoblasts, mesenchymal stem/stromal cells (MSCs), endothelial cells, perivascular cells, immune cells and cells of the nervous system regulate HSCs in their niche. They interact via direct cell-cell interactions as well as secreted factors including cytokines and chemokines [9,10]. The ECM of the bone marrow is a complex composition of collagens, glycoproteins such as fibronectin and laminins, proteoglycans and the glycosaminoglycan hyaluronic acid (HA). The ECM contributes to the regulation of HSC adhesion, migration, proliferation and differentiation by biochemical and biophysical signaling. Biophysical parameters of the ECM influencing HSCs are primarily the nanostructure, the 3D macroscale architecture and the mechanical properties. The bone marrow ECM is highly hydrated, very soft (0.3 to 8 kPa) and the mechanical properties have been reported to influence HSCs and supporting niche cells [8,9]. Translating this knowledge into the bioengineering of clinically relevant *in vitro* models of the HSC niche requires incorporating multiple cell types with precise spatial control in 3D into a matrix that mimics the bone marrow ECM, both in its interaction with cells and its mechanical properties. In this endeavor, bioprinting opens new possibilities by allowing to assemble ECM-mimetic materials in 3D with control over cell positions in all three dimensions. Bioprinting techniques include extrusion-, jetting- and vat-photopolymerization-based bioprinting. Extrusion-based bioprinting refers to the deposition of bioink filaments layer by layer. It is widely accessible, cost-effective and compatible with a wide range of biomaterials [11]. Jetting-based bioprinting involves controlled ejection of small bioink droplets and vat polymerization relies on the photopolymerization of a bioink within a vat [12,13]. While jetting-based bioprinting is most cell-friendly and vat-based bioprinting allows for better resolution and more complex shapes, they are limited by compatible ink formulations and printable cell densities or positioning of different cell types [[11], [12], [13]]. Therefore, although it might be harsher for cells and the achievable resolution is limited, extrusion-based bioprinting best meets the requirements for bioprinting bone marrow-mimetic structures, because it allows bioprinting of hydrogels that mimic the bone marrow ECM in terms of composition and rheological properties, and it allows the positioning of different cell types at relatively high density.

Overall, a hydrogel must fulfill numerous critical criteria to be considered a suitable candidate for extrusion-based 3D bioprinting. It should possess suitable chemical, rheological, mechanical, and biological properties that align with the desired application [14,15]. Hydrogels principally consist of hydrophilic polymers that form networks, allowing them to absorb a significant amount of water [16]. If no hydrogel modifiers are introduced, the final mechanical and biological properties of a 3D-bioprinted hydrogel are determined by the concentration and solvent of the polymer, but predominantly by the intrinsic characteristics of the polymer itself. While synthetic polymers like poly(ethylene oxide), poloxamers, poly(N-isopropylacrylamide) or poly(vinyl alcohol) offer a broad spectrum of diversity and can easily be tailored to produce hydrogels with precise rheological and mechanical properties, they usually show low or negligible biodegradability and limited biocompatibility. In contrast, hydrogels derived from natural polymers, such as hyaluronic acid (HA), collagen, gelatin, silk proteins, methylcellulose, and elastin possess enhanced biocompatibility and biodegradability [17]. Moreover, some of these natural polymers can also be found in the extracellular matrix (ECM) of the HSC niche [8], with particularly HA playing an important role. By interaction with cellular receptors such as CD44 and RHAMM, HA regulates HSC functions including adhesion, migration, proliferation and differentiation [8]. Furthermore, by its ability to retain water, HA determines the volume and hydration of the bone marrow ECM [18] and thus, its physical properties. The bone marrow is a very soft tissue with storage moduli in the range of a few kPa at physiological temperature [19]. The cells of the HSC niche are adapted to their specific ECM and strongly interact with it [8]. Therefore, using natural niche ECM polymers, particularly HA, in HSC niche models is advantageous, as they more accurately mimic the *in vivo* conditions than synthetic polymers. HA fosters improved cellular interactions with the engineered matrix by serving as a natural ligand of HSC receptors and reflecting the biophysical properties of the natural microenvironment. However, the application of natural polymers in 3D bioprinting is constrained by inferior rheological and mechanical characteristics [14]. Addressing this limitation, there is a lot of ongoing research to chemically modify natural polymers, including HA, aiming for 3D-bioprintable hydrogels with improved printing resolution and cytocompatibility [[20], [21], [22], [23]].

HA is a naturally occurring, water-soluble, non-sulfated glycosaminoglycan composed of repeating disaccharide units of D-glucuronic acid and N-acetyl-D-glucosamine [22,24,25]. Due to its biological relevance, HA and its derivatives have been extensively utilized as biomaterials in a wide range of biological and medical applications. HA-based products have subsequently become commercially available, reflecting the widespread adoption of this material [26]. However, hydrogels prepared using unmodified pure HA lack the mechanical and rheological properties necessary for 3D bioprinting, resulting in low shape retention during the printing process. Hydrogels with high HA concentrations are therefore required for such applications, leading to reduced cell viability [27]. To overcome these limitations and combine the benefits of the biologically active HA with those of the easily tunable synthetic polymers, HA can be chemically modified. Depending on the specific requirements, various attributes of HA can be tailored. For instance, its hydrophobicity, biological activity, rheological and mechanical properties can be enhanced to align with the demands of diverse biomedical and biological applications [28]. The modification process typically involves the introduction of new functional groups, small molecules, or polymer chains onto the polymer backbone of HA. This enables the formation of physical and/or covalent chemical bonds between the polymer chains of HA through a chemical crosslinking reaction, or enhances the attractive forces between these chains [27,28]. In the latter scenario, the resulting physical interaction forces between chains are regarded as dynamic non-covalent crosslinking, which can assemble and disassemble reversibly in response to external physical or chemical stimuli. Making use of the dynamic nature of these physical interactions, injectable hydrogels with shear-thinning properties can be prepared for various biomedical applications [29]. Moreover, the immediate reversibility of certain hydrogels upon ceasing the external stimuli qualifies them as suitable candidates for 3D bioprinting. However, to ensure the stability of bioprinted constructs under cell culture conditions, additional strategies involving covalent crosslinking are necessary for bioprinting applications. Consequently, numerous bioprintable hydrogels have been developed, capable of both, covalent and physical crosslinking [30]. Recent developments demonstrate dual crosslinking approaches combining physical interactions (e.g., shear-thinning, guest-host) with covalent chemistries (e.g., disulfide, photo-crosslinking, enzymatic) to optimize HA bioinks for various tissue engineering applications, balancing printability, mechanical robustness, and bioactivity [[31], [32], [33], [34], [35], [36], [37], [38], [39]]. Most of these hydrogels consist of two distinct components that undergo physical crosslinking upon mixing. While such intricate systems allow tight control and tunability of key bioink characteristics including the mechanical properties, degradation rates and biological functions, single component bioinks offer the advantage of simplicity in design and optimization of printing parameters as well as easier quality control and regulatory path due to reduced compositional complexity. Despite these advantages, only few studies have developed single-component HA-based bioinks with HA as a free-standing material. Poldervaart et al. reported bioprinting of 3 % methacrylated high molecular weight HA [40]. Petta et al. presented 3D bioprinting of a tyramine HA derivative by dual enzyme- and light-mediated crosslinking [41,42]. Tavakoli et al. optimized the gelation characteristics of cysteine-modified HA with KI as a catalyst for bioprinting of fine structures [31]. These inks depend on the use of high molecular weight HA [40], which has implications for its biological activity and handling issues [[43], [44], [45]], or on enzyme- or KI-catalyzed pre-gelation of the hydrogel to allow for bioprinting [31,41,42], adding the need of additional processing steps. To enhance and simplify the applicability of HA hydrogels for bioprinting of soft tissues including bone marrow-mimetic constructs, using low molecular weight HA and removing the need of pre-gelation steps would be beneficial.

Towards this goal, the aim of the present study was to develop a minimal, easy to handle, well-defined, biomimetic bioink, to reduce variability while allowing the precise positioning of different cell types within one construct, thereby serving as an enabling platform to create customizable 3D *in vitro* models of soft tissues such as the bone marrow HSC niche. For this purpose, a novel single-component, cytocompatible and 3D-bioprintable hydrogel derived from chemically modified low molecular weight HA was developed, allowing covalent and physical crosslinking. Through a straightforward one-pot synthesis, small alkyl chains and functional methacrylamide groups were introduced onto the polymer backbone, enhancing hydrophobic interactions and enabling light-induced crosslinking. This ensured high shape fidelity and stability of bioprinted constructs under cell culture conditions. As a first proof-of-principle for the suitability of this hydrogel as a biomimetic bone marrow analogue and hence for the usage in HSC niche models, an MSC and an HSC model cell line (iMSC#3 cells and KG-1a cells, respectively) were bioprinted in it. The cells were either directly incorporated within the HA-based hydrogel and bioprinted with the gel or injected into pre-printed hydrogel structures before covalent crosslinking, taking advantage of the self-healing properties of the hydrogel (two-step bioprinting). Thus, in advancing previous HA-based bioinks, we introduce a single-component, additive-free bioink utilizing a low molecular weight HA derivative as self-supporting material. This new ink simplifies bioprinting of HA-based bioinks by eliminating the need for multiple component formulation and pre-gelation processing steps. Moreover, it enhances versatility across various cell types supporting not only cell encapsulation but also post-printing cell injection, making it suitable for both, direct extrusion-based as well as two-step bioprinting. Combined with its excellent rheological, shear-thinning, self-healing and cytocompatible characteristics, these advantages render the new hydrogel ideal for developing easily adaptable 3D *in vitro* models of the HSC niche, paving the way for advanced biomedical applications.

## 2 Materials and methods

Sodium HA salts (MW = 40–50 kDa, and 80–100 kDa, in the following indicated by the index values 40 or 80, respectively) were purchased from Carbosynth Limited and stored at −20 °C. Tetrabutylammonium hydroxide solution, (TBA, 40 %(w/v) in water), hexadecylamine (HD, 98 %), dodecylamine (DD, ≥99 %), anhydrous N,N-dimethylformamide (DMF), Bis(4-nitrophenyl)-carbonate (4-NPBC), Dowex™ 500WX8-200 ion-exchange resin, N-(3-aminopropyl)-methacrylamide hydrochloride (APMA, 98 %), lithium-phenyl-2,4,6-trimethylbenzoylphosphinate (LAP, ≥95 %), α-hydroxy-4-(2-hydroxyethoxy)-α-methylpropiophenone (Irgacure 2959, 98 %) were bought from Sigma-Aldrich and used as received. Sodium hydroxide, acetone, diethyl ether, sodium chloride and dialysis membrane (regenerative cellules) were purchased from Carl Roth. Tetrabutylammonium salts of HA (HA-TBA) were prepared following the procedure described in detail by Loebel et al. [46].

### 2.1 Synthesis of hyaluronic acid–hexadecylamine (HA-HD) and hyaluronic acid–dodecylamine (HA-DD) derivatives

HA-TBA was chemically modified with either HD or DD hydrophobic short chains by following the procedure described by Bongiovì et al. with some modifications (Fig. S1) [47]. In a typical experiment, 8 g of HA-TBA were added to a 500 mL three-necked round bottom flask, previously oven-dried. The flask was purged with nitrogen several times before the addition of approximately 120 mL of anhydrous DMF to dissolve the polymer overnight. The next day, the solution was heated to 40 °C and a suitable amount of 4-NPBC dissolved in anhydrous DMF was added dropwise to the reaction solution, followed by stirring at this temperature for 4 h. The reaction temperature was then raised to 60 °C, and an excess amount of HD or DD, dissolved in 40 mL of anhydrous DMF, was added dropwise. The reaction mixture was left stirring under nitrogen overnight at 60 °C. The following day, the heating source was removed and 3 mL of a saturated aqueous NaCl solution was added under stirring in order to exchange the TBA cations with sodium cations. The viscosity of the solution significantly increased upon addition of the NaCl solution, requiring manual mixing to ensure complete cation exchange. The resulting product was then precipitated by adding it to a diethyl ether/chloroform 1:1 (v/v) solution. After precipitation, the product was washed with an ethanol/water 9:1 (v/v) solution, followed by pure ethanol. The polymer was further purified by dialysis against water for 24 h and then freeze-dried for 3 days, resulting in a solid light yellowish product. The prepared polymer was finally stored at −20 °C. The yield of the different prepared polymers ranged between 70 and 80 %(w/w). The modification ratio was determined using ^1^HNMR spectroscopy (Fig. S2) and is given as index values following the abbreviations HD or DD. ^1^HNMR spectra were recorded using a Bruker Ultrashield 500 MHz Avance IIIH, Bruker Ascend 600 MHz Avance Neo or Bruker Ascend 400 MHz Avance III.

### 2.2 Synthesis of hyaluronic acid–hexadecylamine-N-(3-aminopropyl)-methacrylamide (HA-HD-MA) and hyaluronic acid–dodecylamine-N-(3-aminopropyl)-methacrylamide (HA-DD-MA) derivatives

**Fig. 1:**
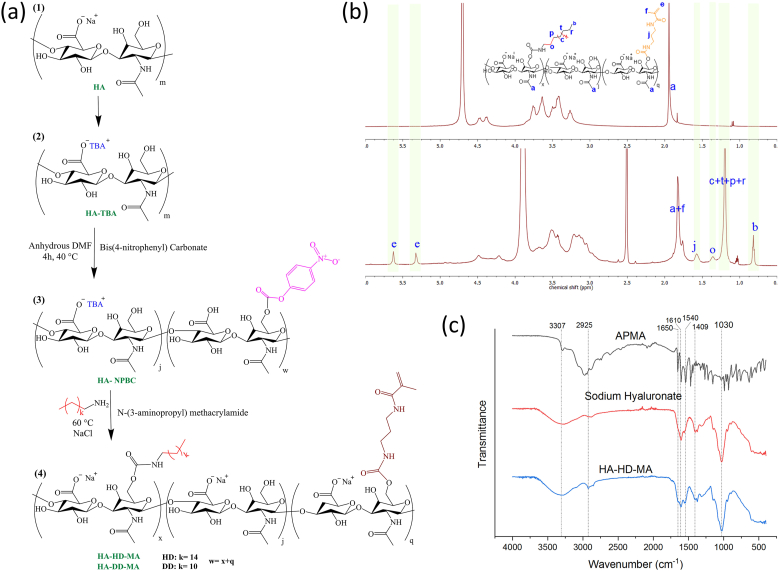
(a) Synthesis steps to chemically modify sodium hyaluronate. (b) Comparison between the ^1^HNMR spectra of sodium hyaluronate (top) and the modified polymer (bottom). (c) ATR spectra of APMA, sodium hyaluronate and HA-HD-MA.

The modification reaction for the photo-crosslinkable hydrogel was carried out similarly to that described for the preparation of HA-HD and HA-DD polymers. The only difference was the addition of APMA during the step of HD or DD addition (Fig. 1(a)). The successful synthesis of the polymers was confirmed by ^1^HNMR and ATR spectroscopy (Fig. 1(b) and (c)). The methacrylamide (MA) modification ratio was again determined using ^1^HNMR spectroscopy (Fig. 1(b)). The ATR spectra were recorded with a Shimadzu IRAffinity-1S with quest ATR unit. The MA modification ratio is indicated by an index number following MA in the nomenclature of the synthesized polymers.

### 2.3 Hydrogel preparation

The standard procedure for preparing a 4 %(w/w) hydrogel was as follows: 60 mg of the modified polymer were added to a 2.0 mL polypropylene reaction tube, followed by the addition of 1.44 mL of PBS buffer. Furthermore, 0.1 mL of Irgacure 2959 solution (15 %(w/v) in ethanol) were added to the tube. The tube was then placed in a thermostatic shaker at 45 °C for approximately 48 h. To ensure homogeneity of the prepared hydrogel, the tube was opened several times during the shaking process and its content was mixed manually using a small spatula. Subsequently, the hydrogel was centrifuged to remove any formed bubbles. Finally, the hydrogel was stored in the dark at 4 °C.

### 2.4 Qualitative cohesion test

To test the self-healing properties of 6 %(w/w) HA_80_DD_15_MA_13_ hydrogels, two hydrogels were prepared separately from each other and were stained with blue and red food coloring to be distinguishable. The interfaces of these hydrogels were brought into contact for 10 min by placing them on top of each other and the gels were subsequently UV-crosslinked for 10 min. External tensile forces were applied using forceps to assess whether the gels would separate again.

### 2.5 Rheological characterization

The rheological properties of the tested hydrogels were investigated using small amplitude oscillatory shear (SAOS) on a modular MCR 302 rheometer (Anton Paar, Graz, Austria). This rheometer was equipped with a UV light source (365 nm, LED lamp with 20 mW/cm^2^, located under the rheometer's lower plate, Delolux 80, Delo, Germany), a removable aluminum plate with a diameter of 20 mm (upper plate) and a temperature-controlled water bath that circulated water to regulate the temperature of the rheometer's lower plate. Unless otherwise specified, all experiments were conducted at 37 °C using 4 %(w/w) hydrogels and were repeated three times. Throughout the experiments, a distance of 0.5 mm was maintained between the upper and lower plate to confine the measured samples, while a water trap was employed to cover any sample gap, thereby preventing sample dehydration.

The viscosity of the uncured hydrogels was determined via rotational viscosimetry using the same equipment, with the shear rate varying from 1 to 100 s^−1^.

Cyclic strain time sweep experiments involved alternation between low and high strain regions. A low-strain region with 0.1 % deformation was applied for 2 min, followed by a high-strain region where 500 % deformation was applied for 1 min. This cycle was repeated five times for each test. The healing efficiency after each deformation cycle was calculated via the ratio of the storage moduli G’ after deformation and the initial value as follows:

$$Healing\;Efficiency=\frac{G_{healed}^{\prime }}{G_{initial}^{\prime }}\cdot 100\;\%$$

The kinetics of the crosslinking reaction were studied by initially measuring the rheological properties of the uncured polymer for 5 min. Subsequently, the polymerization process was initiated by illuminating the hydrogel with UV light for a predetermined period of time (2–10 min), followed by further measurements in the dark for 10 min.

Mesh sizes were estimated from the storage moduli G’ of photo-crosslinked hydrogels according to Refs. [48,49] with the Avogadro constant *N*_*A*_, the molar gas constant *R* and the temperature *T* via the following formula:

$$\xi ={\left(\frac{G^{\prime }N_{A}}{RT}\right)}^{-1/3}$$

### 2.6 Swelling analysis

The swelling behavior of the photo-crosslinked HA_40_HD_15_MA_18_ and HA_80_DD_15_MA_13_ hydrogels (4 %(w/w), 0.05 %(w/w) LAP) was investigated gravimetrically. Gels in the shape of a cylinder were extrusion-printed into 35 × 10 mm cell culture dishes (Greiner Bio-One) and the original weight of the gels was determined (w_original_). The gels were then immersed in 3 mL Dulbecco's Phosphate Buffered Saline (PBS) (Sigma-Aldrich) and weighed after 1 min, 5 min, 10 min, 30 min, 1 h, 2 h, 3 h, 4 h and 24 h. For this purpose, the PBS was removed and the mass of the swollen gels was noted (w_swollen_). 6 gels were printed and weighed for each variant. The weight difference of the swollen gels relative to the original weight was determined. The swelling ratio was calculated using the following equation:

$$Swelling\;ratio=\frac{\left(W_{swollen}-W_{original}\right)}{W_{original}}\;\cdot 100\;\%$$

### 2.7 3D printing of HA-based hydrogels

A multi-head 3D bioprinter (3D Discovery™ Evolution, RegenHU, Switzerland) was employed to assess the 3D printability of various hydrogels as well as for 3D bioprinting. For each test, a 3 mL dispensing cartridge (Nordson EFD, Feldkirchen, Germany) equipped with a white syringe barrel (SmoothFlow, Nordson EFD) and a precision tip with an inner diameter of 0.26 mm was filled with an adequate amount of the hydrogel under evaluation. All 3D prints were conducted at room temperature.

To generate the G-code for the scaffold with rectangular shape, the BIOCAD software (RegenHU) was utilized. If not stated differently, the distance between printed filaments was set to 1.5 mm, with a layer thickness of 0.29 mm. The printing speed was adjusted to 40 mm s^−1^. Each printed layer underwent an initial crosslinking process lasting 3 min using the UV light curing kit integrated into the 3D printer (365 nm with an optical power of 360 mW). Subsequently, further curing was carried out for 10 min post-printing using a stronger UV lamp (365 nm equipped with two 8 W LED lamps).

#### 2.7.1 Printability analysis

The printability of the bioinks was semi-quantitatively examined by determining the printability (Pr), uniformity (U) and strand width. 4 %(w/w) hydrogels of the differently modified HA-based polymers were prepared in PBS and 0.05 %(w/w) LAP was added to the MA-modified variants. The gels were stained with blue food colorant (Unsere Lebensmittel Farben, RUF). Square-shaped grids with a side-length of 1 cm, 2 layers and an inter-filament distance of 2 mm were extrusion-printed with a layer thickness of 0.15–0.2 mm using a 3 mL amber cartridge with a white syringe barrel and a 0.33 mm blunt precision tip. The gels were printed onto microscopy slides. The MA-modified variants were additionally photo-crosslinked with blue light (420 nm) with a blue light lamp (Prizmatix) for 3 min. The printed grids were imaged with a stereomicroscope (Stemi 508, Zeiss, Germany). The images were analyzed using Fiji [50] and Zen 3.3 (Carl Zeiss GmbH). The printability factor (Pr) was determined by measuring circularity (C), perimeter (L) and area (A) of the inner 9 pores of n = 3 printed grids per polymer. Pr was calculated according to Ouyang et al. [51] using the following formula:

$$\mathrm{Pr}=\frac{\pi }{4}\cdot \frac{1}{C}=\frac{L^{2}}{16A}$$

The uniformity (U) was evaluated as the ratio of the measured length (L_M_) and the theoretical length (L_T_) of strands in the second layer of the printed grids [52].

$$U=\frac{L_{M}}{L_{T}}$$

Strand width was determined by measuring the diameter of central parts of the strands in the second layer of the printed grids.

#### 2.7.2 3D bioprinting of 2 %(w/w) hydrogels with encapsulated cells

For 3D bioprinting of the designed hydrogels including encapsulated cells, the required amount of the polymer HA_40_HD_15_MA_18_ or HA_80_DD_15_MA_13_ was sterilized under UV light for 15 min and then dissolved at 2 %(w/w) in RPMI-1640 medium including 20 %(v/v) fetal bovine serum (FBS, Sigma-Aldrich), 100 U/mL penicillin and 100 μg/mL streptomycin. As photoinitiator, 0.05 %(w/w) LAP was added. The mixture was vigorously shaken in the dark on a thermostatic shaker at 50 °C for at least one day and stored at 4 °C afterwards.

**Fig. 2:**
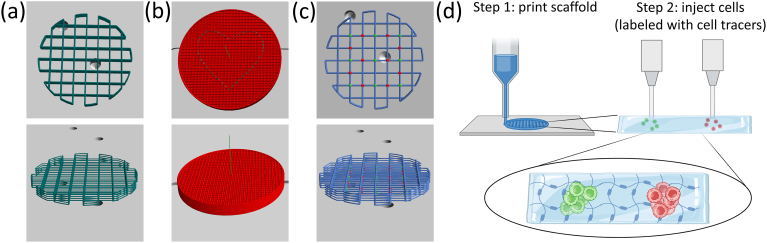
3D designs for bioprinting with HA_40_HD_15_MA_18_ and HA_80_DD_15_MA_13_ in top (upper row) and side view (lower row). (a) 3D design for bioprinting of 2 %(w/w) hydrogels with encapsulated cells (direct bioprinting) as grid structure (dark green lines). (b) Scheme of a construct for which a 4 %(w/w) hydrogel is deposited first as a dense grid (red lines) to yield a compact cylinder and cells are injected in a heart shape (grey lines) in a second bioprinting step (two-step bioprinting). (c) 3D design for bioprinting of a 4 %(w/w) hydrogel in a grid shape first (blue lines) and inserting KG-1a (green dots) and iMSC#3 cells (red dots) into the nodes of the grid in a second step (two-step bioprinting). (d) Schematic drawing illustrating the two-step bioprinting process of extruding the hydrogel in the first step and injecting different cell types in the second step into the hydrogel, yielding cell assemblies positioned within the hydrogel structure. Schematic drawing in (d) was created in BioRender. Lee-thedieck, C. (2025) https://BioRender.com/zz6g51v.

Before usage, the hydrogels were prewarmed to 37 °C and mixed again by pipetting up and down with a 1000 μL piston pipette with a cut tip. Subsequently, iMSC#3 and KG-1a cells (see section 2.8) were harvested, centrifuged down and directly resuspended in the 2 %(w/w) hydrogels to yield a final concentration of 5x10^6^ or 1x10^7^ cells mL^−1^. The applied cell concentrations are given in the respective figure legends. A sterile 3 mL dispensing cartridge (Nordson EFD) with a white syringe barrel (SmoothFlow, Nordson EFD) and a precision tip with an inner diameter of 0.33 mm was filled with the gel/cell mixture. Applying a pressure of 55 kPa, a grid in the shape of a cylinder (Fig. 2(a)) was bioprinted directly into a 6-well plate with a feedrate of 60 mm s^−1^. The grid consisted of 10 layers with a layer thickness of 0.15 mm and an inter-filament distance of 2 mm. After every two layers, the bioprinted filaments were irradiated by UV light (UV lamp of the bioprinter) for 30 s. At the end of the bioprinting process, the entire grid was illuminated with blue light (420 nm, blue light lamp from Prizmatix) for additional 3 min. Subsequently, 3 mL of alpha MEM with 10 %(v/v) FBS, 100 U/mL penicillin and 100 μg/mL streptomycin for iMSC#3 and RPMI-1640 with 20 %(v/v) FBS, 100 U/mL penicillin and 100 μg/mL streptomycin for KG-1a cells were added to the well and the samples were incubated overnight at 37 °C. On the following day, a live-dead staining (see section 2.10) was performed for initial bioprintability assessment. Long-term cell viability was tested for iMSC#3 and KG-1a cells in the HA_80_DD_15_MA_13_ variant. For these tests, bioprinting was performed as described above with the following alterations: Grids were bioprinted into a 12-well plate (Greiner Bio-One) with a feedrate of 25 mm s^−1^. The grid consisted of 10 layers with a layer thickness of 0.075 mm and an inter-filament distance of 2 mm. 1 mL cell culture medium was used per well and media were exchanged every 2–3 days. Live-dead staining (see section 2.10) for long-term cell viability studies was performed after 3, 7 and 14 days.

#### 2.7.3 Two-step 3D bioprinting of cells into a 4 %(w/v) hydrogel

For 3D bioprinting with 4 %(w/w) hydrogels, the polymers HA_40_HD_15_MA_18_ or HA_80_DD_15_MA_13_ were sterilized under UV light for 15 min and then dissolved in RPMI-1640 medium containing 20 %(v/v) FBS, 100 U/mL penicillin, 100 μg/mL streptomycin and 0.05 %(w/w) LAP as photoinitiator. After shaking them strongly on a thermostatic shaker at 50 °C for at least one day in the dark, they were transferred into a sterile 3 mL dispensing cartridge (Nordson) with a white syringe barrel (SmoothFlow, Nordson) and a precision tip with an inner diameter of 0.33 mm. KG-1a and/or iMSC#3 cells were harvested, centrifuged down and resuspended in their corresponding cell culture medium including penicillin and streptomycin (see section 2.8).

As a first approach, the 4 %(w/w) hydrogels were extruded into a 6-well plate as flat compact gel cylinders with 10 completely filled layers (inter-filament distance 0.5 mm, layer thickness 0.17 mm, feedrate 30 mm s^−1^, air pressure: 250–300 kPa). Into this, cells were injected in the shape of a heart (Fig. 2(b)). To do so, the cell suspension (KG-1a cells with 3x10^7^ cells mL^−1^ or iMSC#3 cells with 3.75x10^7^ cells mL^−1^) was filled into the sterile 3 mL cartridge of a cell-friendly inkjet printhead of the bioprinter equipped with a contact dispensing microvalve (MVC-D0.3S0.06, nozzle diameter 0.3 mm, stroke 0.06 mm, regenHU) and a metal needle with an inner diameter of 0.3 mm (NCF-D0.3L6, external diameter 0.5 mm, length 6 mm, regenHU). In 4 layers with an inter-layer distance of 0.2 mm, the cells were then bioprinted into the pre-printed hydrogel (air pressure: 11 kPa, dosing distance: 0.2 mm, valve opening time: 0.5 ms, valve closing time: 3 ms, feedrate: 5 mm s^−1^). Supplementary video 1 shows the extrusion of a hydrogel, the subsequent bioprinting of the cells into the gel and the self-healing of the hydrogel upon the cell injection (supplementary information, video 1). After completion of the bioprinting process, the hydrogel was crosslinked by irradiation with blue light (420 nm) for 5 min. The combination of 0.05 % LAP and blue light irradiation for 5 min was chosen as it was shown to result in high cell viability [53,54] and at the same time led to efficient photo-crosslinking of the hydrogels. Finally, 3 mL of the respective medium were added to the well and the samples were cultivated at 37 °C overnight, followed by a live-dead staining on the next day (see section 2.10).

As a second approach, a grid structure was first extruded with the 4 %(w/w) hydrogels into a 6-well plate (air pressure: 250–300 kPa, feedrate: 30 mm s^−1^). The grid consisted of 10 layers with a layer thickness of 0.17 mm and an inter-filament distance of 2 mm. Into the nodes of this grid, KG-1a cells and iMSC#3 cells (both in a concentration of 10^7^ cells mL^−1^), which were stained with cell tracking dyes beforehand (see section 2.9), were injected in an alternating pattern. Per node, one cell type was placed as dots in 5 layers on top of each other with an inter-layer distance of 0.2 mm (Fig. 2(c and d)). KG-1a cells were bioprinted with the cell-friendly printhead using the same parameters as already described, while iMSC#3 cells were filled into a sterile modified 0.1 mL Hamilton syringe (regenHU) equipped with a precision tip (0.33 mm inner diameter) and fitting the high precision plunger dispenser printhead of the bioprinter. The cells were injected with a feedrate of 25 mm s^−1^ and a volume flow rate of 0.1 μL s^−1^. After injection of the cells, the samples were illuminated with blue light (420 nm) for 5 min to crosslink the hydrogel. Subsequently, 3 mL of RPMI-1640 medium including 20 %(v/v) FBS, 100 U/mL penicillin and 100 μg/mL streptomycin were added to the well. On the same day, the samples were imaged via confocal laser-scanning microscopy (LSM 980, Carl Zeiss, Oberkochen, Germany). Images showing the entire hydrogel were taken with a 2.5x objective using the *tiles and positions* option of the microscopy software. This method involves capturing overlapping images and employing software-based assembly afterwards to generate a unified image. For a detailed orthogonal view of adjacent nodes, a z-stack was recorded using a 10x objective in conjunction with the *tiles and positions* mode.

### 2.8 Cell culture

Cells were always cultivated in an incubator at 37 °C in a humidified atmosphere with 5 % CO_2_. The leukemic cell line KG-1a was maintained in suspension in RPMI-1640 with L-glutamine and sodium bicarbonate (Sigma-Aldrich) and 20 %(v/v) FBS. For 3D bioprinting experiments, 100 U/mL penicillin (Sigma-Aldrich) and 100 μg/mL streptomycin (Sigma-Aldrich) were added to the medium. The adherent mesenchymal stem/stromal cell line iMSC#3 [55] was cultured in alpha MEM with stable glutamine (P04-21250, PAN-Biotech, Aidenbach, Germany), 10 %(v/v) FBS and 2 μg/mL puromycin (Sigma-Aldrich). For 3D bioprinting experiments, puromycin was replaced by 100 U/mL penicillin and 100 μg/mL streptomycin.

### 2.9 Cell staining with cell tracking dyes

To be able to visualize the cells via confocal laser scanning microscopy after bioprinting and to distinguish between KG-1a and iMSC#3 cells, they were stained with two different cell tracking dyes the day before bioprinting.

KG-1a cells were stained with CytoPainter Cell Proliferation Staining Reagent – Green Fluorescence (ab176735, Abcam, Cambridge, UK). To prepare the staining solution 8 μL of a 500X stock solution of the CytoPainter (prepared following the manufacturer's instructions) were added to 4 mL Roti®Cell Hanks' BSS (Carl Roth, Karlsruhe, Germany) containing 20 mM HEPES (Sigma-Aldrich). 4x10^6^ cells were centrifuged down, resuspended in the staining solution and transferred into a T25 suspension flask. After incubating for 30 min at 37 °C, the cells were centrifuged down, resuspended in RPMI-1640 with 20 %(v/v) FBS and kept overnight in the incubator at 37 °C to be used for 3D bioprinting the next day.

iMSC#3 cells were stained with CellTracker™ Deep Red dye (ThermoFisher Scientific). The dye was dissolved following the manufacturer's instructions to obtain a 1 mM stock solution, which was then diluted with alpha MEM to receive a 25 μM staining solution. The medium of iMSC#3 cells growing in a monolayer in a T75 cell culture flask was replaced by 1.5 mL staining solution. Subsequently, the cells were incubated at 37 °C for 30 min. Afterwards, the staining solution was removed and the cells were maintained in alpha MEM with 10 %(v/v) FBS and 2 μg/mL puromycin in the incubator overnight to be used for 3D bioprinting (see section 2.7.3) the following day.

### 2.10 Live-dead staining

On day 1, 3, 7 and 14 after 3D bioprinting (see sections 2.7.2 and 2.7.3), the viability of the cells in the HA-based hydrogels was examined via live-dead staining. Therefore, a staining solution was prepared by dissolving 1 μM Calcein-AM (marking living cells, Sigma-Aldrich), 10 μM propidium iodide (staining dead cells, Sigma-Aldrich) and 20 μg/mL Hoechst 33342 (staining all nuclei, Sigma-Aldrich) in PBS. The samples were incubated for 30 min at 37 °C, the staining solution was exchanged with PBS and afterwards directly imaged with a confocal laser-scanning microscope (LSM 980, Carl Zeiss) using 2.5x and 10x objectives. Additionally, entire hydrogels were captured with the *tiles and positions* feature of the microscopy software and a 2.5x objective and assembled in the software to a joint image afterwards. For long term cell viability assessment, the hydrogels were imaged using the 10x objective. Pictures of different parts of the gels were taken and the total cell number (Hoechst 33342 stained nuclei) and the number of dead cells (propidium iodide stained cells) were determined with Fiji [50]. Using these numbers, the percentage of living cells was calculated.

To demonstrate cell proliferation within the hydrogels, diameters of cell clusters were determined on day 1, 3, 7 and 14 after bioprinting.

### 2.11 Quantitative RT-PCR

RNA was extracted from KG-1a and iMSC#3 cells cultured for 7 and 14 days in the bioprinted hydrogels (which were preserved after culture in RNAprotect (Qiagen) and stored at −80 °C) using Trizol (Sigma Aldrich) and chloroform (Sigma Aldrich) in combination with the RNeasy Micro Kit (Qiagen). KG-1a and iMSC#3 cells from standard maintenance cell cultures served as controls. The Maxima First Strand cDNA Synthesis Kit for RT-qPCR with dsDNase (Thermo Fisher Scientific) was used to transcribe cDNA from the extracted RNA. From all samples, 12.5 ng cDNA were used as templates for the qPCR which was carried out using the Luna Universal qPCR Master Mix (New England Biolabs) with primers listed in Supplementary Table S1 on the qTOWER^3^ G (Analytik Jena). qPCR was performed for 45 cycles. Each sample and target gene were analyzed in technical triplicates. The fold change was calculated using the 2^−ΔΔCt^ method.

## 3 Results and discussion

Hydrogels derived from HA offer a versatile platform for various applications in biomedicine and tissue engineering due to their tunable mechanical properties, biocompatibility and resemblance to the ECM [22,28]. By introducing hydrophobic alkyl chains, namely hexadecylamine (HD) or dodecylamine (DD), and functional methacrylamide groups (MA), we further tailored the HA backbone to meet our specific needs for developing a 3D matrix suitable for *in vitro* models of the HSC niche.

### 3.1 Synthesis and characterization of hydrophobically modified HA-based polymers

As basis for our HA-based hydrogels, HA with a molecular weight of 40 or 80 kDa (HA_40_ or HA_80_) was used, indicated with an index number following HA in the polymer nomenclature. In the modification reaction, the sodium cation of HA was initially substituted with the TBA cation to make HA soluble in DMF. In order to streamline the synthesis procedure, the modification reaction was conducted in a one-pot manner, following a two-step synthetic route (Fig. 1(a)). In the first step, a portion of the primary hydroxyl groups of HA-TBA was modified using 4-NPBC. The modification ratio in this step equaled the sum of the targeted modification ratios of alkyl chains and, if included, functional MA groups. The modification ratios of the different functional groups (DD or HD and MA) are given as indices in the polymer name (e.g., HA_40_HD_15_MA_18_ refers to 40 kDa HA with modification ratios of 15 % HD and 18 % MA). A three-fold molar equivalent excess of each reagent was added in the subsequent step to ensure complete substitution of 4-NPBC groups by alkyl and APMA groups. The advantages of introducing hydrophobic alkyl chains over supramolecular guest-host modifications, which have also been successfully used to generate injectable dual-crosslinkable HA-based polymers [30], include a significantly shorter synthesis procedure and the necessity of only one type of polymer. In contrast, guest-host interactions require two different polymers – one functionalized with guest moieties and the other with host moieties.

To confirm the successful synthesis of the polymers, ^1^HNMR and ATR spectroscopy were performed (exemplary spectra in Fig. 1(b) and (c)). A comparison of the ^1^HNMR spectrum of HA-HD-MA with that of pure HA shows the presence of two new peaks at chemical shifts of 5.33 and 5.61 ppm, belonging to the characteristic vinyl group of APMA, while the peaks at 0.82 and 1.19 ppm can be assigned to the introduced HD alkyl chains (Fig. 1(b)). In the ATR spectrum of APMA, the absorption band between 3500 and 2800 cm^−1^ is associated with NH stretching vibrations, while the peak at 1650 cm^−1^ is attributed to C=O stretching vibrations, which are also visible in the spectrum of HA-HD-MA. The strong band at 1610 cm^−1^ corresponds to the stretching vibration of the alkene group C=C [56]. Because the lateral band is overlapped in the spectrum of the final product by bands at 1610, 1616 and 1540 cm^−1^, attributed to amide I, carboxyl groups and amide II of sodium hyaluronate, respectively [34,57], it cannot be characterized in the final product (Fig. 1(c)). In conclusion, the successful synthesis of the polymers can be validated with ^1^HNMR and ATR spectroscopy.

To assess the impact of introduced alkyl side chains on the hydrogel-forming capability of the synthesized polymers, solutions of polymers derived from HA_40_ or HA_80_ with varying modification ratios of HD or DD were prepared in PBS at a concentration of 4 %(w/w) and examined via inversion tests (Table 1). Modification of HA_40_ with low HD modification ratios (≤ 5 mol%) results in polymers only forming flowable liquids while those with modification ratios between 10 and 15 mol% form stable hydrogels. However, if the modification ratio is ≥ 20 mol%, the resulting polymers are insoluble in PBS. Conversely, using the shorter DD alkyl chain requires higher modification ratios compared to HD in order to achieve stable hydrogel formation at a concentration of 4 %(w/w). For instance, while HA_40_HD_10_ and HA_40_HD_15_ form stable hydrogels, HA_40_DD_10_ and HA_40_DD_15_ yield flowable liquids. The longer HD chains induce stronger hydrophobic interactions, thereby forming more robust physical crosslinks among the polymer chains in PBS, consequently promoting hydrogel formation [58,59]. Additionally, hydrogel formation is already achievable at lower modification ratios if HA_80_ is used instead of HA_40_. This is evident, for example, when comparing the behavior of the 4 %(w/w) solution of HA_40_DD_10_, which forms a flowable liquid, with that of HA_80_DD_10_, which is capable of hydrogel formation. This observation suggests that increasing the molar mass of HA enhances hydrogel formation when dissolved in PBS, due to network strengthening caused by the aggregation of HA chains through hydrophobic interactions and intermolecular hydrogen bonds [43,60].

**Table 1:** Chemical compositions of the synthesized hydrophobically modified HA-based polymers and their hydrogel-forming properties as 4 %(w/w) solutions in PBS (studied via inversion tests). HA: hyaluronic acid, HD: hexadecylamine, DD: dodecylamine, MA: methacrylamide.

Sample	HA molar mass [kDa]	Modification ratio [mol%]		Solution in PBS [4 %(w/w)]
		HD or DD	MA	
HA_40_HD_5_	40	5	–	flowable liquid
HA_40_HD_10_	40	10	–	hydrogel
HA_40_HD_15_	40	15	–	hydrogel
HA_40_HD_20_	40	20	–	insoluble
HA_40_DD_10_	40	10	–	flowable liquid
HA_40_DD_15_	40	15	–	flowable liquid
HA_40_HD_15_MA_18_	40	15	18	hydrogel
HA_80_HD_10_	80	10	–	hydrogel
HA_80_HD_20_	80	20	–	Insoluble
HA_80_DD_10_	80	10	–	hydrogel
HA_80_DD_25_	80	25	–	hydrogel
HA_80_DD_15_MA_13_	80	15	13	hydrogel

While some of the polymers described so far are able to reliably form hydrogels and may serve as suitable candidates for injectable hydrogels, none of these can be used for 3D bioprinting. This is because the physical crosslinking of the polymer chains is not stable enough when the hydrogel is exposed to an aqueous environment as needed in cell or tissue culture. Consequently, the hydrogel would gradually dissolve over time when being exposed to cell culture medium, which is essential for providing adequate nutrient supply during further cell culturing. To improve the stability of 3D-bioprinted hydrogels in aqueous cell culture media, HA was further functionalized with APMA to introduce MA groups to allow light-induced chemical crosslinking. For this purpose, two polymers were synthesized, of which hydrogel formation was expected. The resulting polymers, HA_40_HD_15_MA_18_ and HA_80_DD_15_MA_13_, are capable of forming stable hydrogels (Table 1). Even at polymer concentrations of 2 %(w/w) in PBS, the polymer solutions yield soft but non-flowing hydrogels. These results indicate relatively strong internal cohesion forces within the tested hydrogels, which are necessary for maintaining the shape fidelity of a 3D-bioprinted filament, preventing the bioprinted construct from losing its shape prior to chemical crosslinking.

### 3.2 Rheological properties

The before suggested cohesion forces also become apparent qualitatively when the interfaces of two separately prepared hydrogels are brought into contact for 10 min. After subsequent UV crosslinking for 10 min, the hydrogels cannot be pulled apart, showing the formation of a unified intact hydrogel (Fig. S3). Such self-healing properties of the hydrogel are particularly advantageous during 3D printing as they assure the connection of the different layers at the intersection points [61]. To further test the suitability of the generated hydrogels for extrusion-based 3D bioprinting also quantitatively, their rheological properties were analyzed, as they provide valuable information about the characteristics of the hydrogel and give an idea about the environment and mechanical forces, which living cells are possibly exposed to before, during and after the bioprinting process [62].

**Fig. 3:**
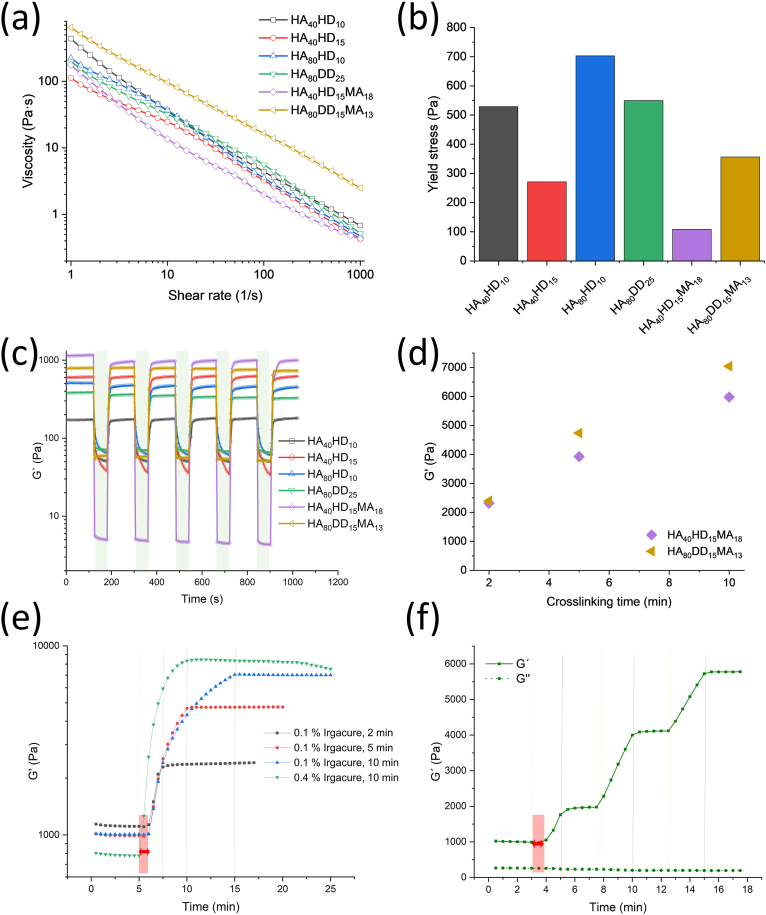
Rheological properties and relaxation behavior of HA-based hydrogels. Using various 4 %(w/w) HA-based hydrogels, viscosity (a) and yield stress (b) measurements as well as cyclic strain time sweep experiments with an alternating strain of 0.1 % (low, unshaded area) and 500 % (high, shaded area) (c) were performed. (d) G′ at 1 % strain after 2, 5 and 10 min UV exposure for 4 %(w/w) HA_40_HD_15_MA_18_ (violet diamonds) and HA_80_DD_15_MA_13_ (yellow triangles) hydrogels. (e, f) Studies of the kinetics of the photo-crosslinking reaction for 4 %(w/w) HA_80_DD_15_MA_13_ with varying UV exposure times. The curing lag time is indicated with a red shading and a red double-pointed arrow while vertical lines indicate the beginning (yellow dotted lines) and end (purple dashed lines) of the UV irradiation.

The physically (non-covalently) crosslinked hydrogels display storage moduli between 232 Pa and 772 Pa for the different hydrophobically modified HA hydrogels. The polymers with additional MA modification, HA_40_HD_15_MA_18_ and HA_80_DD_15_MA_13_, display storage moduli of 1070 Pa and 742 Pa, respectively (Fig. S4). Furthermore, rheology experiments reveal a significant reduction of viscosity with increasing the applied shear rate, thereby showing shear-thinning behavior of all tested hydrogels (Fig. 3(a)). This property is beneficial for 3D bioprinting of cell-laden hydrogels as it reduces the necessary applied pressure and therefore the induced shear forces during the extrusion process. Accordingly, cell viability is enhanced [63]. Furthermore, also the natural bone marrow shows decreasing viscosity with increasing shear rates [19,64]. At high shear rates, HA_80_DD_15_MA_13_ exhibits the highest viscosity, whereas HA_40_HD_15_MA_18_ shows the lowest value compared to the other hydrogels, which seems to correlate with the molecular weight of the base polymer. Another parameter to assess the shear-thinning behavior of hydrogels and hence their suitability for 3D bioprinting is the yield stress, which is defined as the minimum shear stress required for a hydrogel to begin flowing [65]. In bioprinting, this value indicates the amount of pressure needed for the extrusion process. Yield stress is determined from the storage modulus – shear stress diagram by identifying the point of intersection between the linear tangents established at the plateau region and the region where the storage modulus decreases (Fig. S5). Evaluating the yield stress values of the studied hydrogels, HA_40_HD_15_MA_18_ shows the smallest yield stress, followed by HA_40_HD_15_ and HA_80_DD_15_MA_13_ (Fig. 3(b)).

While on the one hand shear-thinning properties are of great importance in 3D bioprinting to liquefy and smoothly extrude hydrogel filaments, it is on the other hand essential for an improved shape fidelity in a way that, after extrusion, the hydrogel rapidly returns to its initial mechanical state, i.e. before applying shear forces. In this way, the cylindrical shape of the extruded strands can be retained and coalescence between these filaments can be prevented. The ability to regain the mechanical state was analyzed by cyclic strain time sweep experiments with alternating strains of 0.1 % and 500 % (Fig. 3(c)). If a high strain ratio is applied, imitating the occurring strain change during the extrusion process, an abrupt drop in the storage moduli of all tested hydrogels can be observed. Upon reducing the applied strain ratio again, the storage moduli of all examined hydrogels immediately increase. The hydrogels prepared from the photo-crosslinkable polymers HA_40_HD_15_MA_18_ and HA_80_DD_15_MA_13_ show the highest storage moduli at low strain while exhibiting the smallest values compared to the other polymers at high strain and thus the most advantageous rheological properties for 3D bioprinting. Throughout all five measuring cycles, the rapid recovery of mechanical properties upon high strain can consistently be observed in all hydrogels, with the storage moduli returning to their initial levels, only showing a slight decrease after the first cycle. Thus, with healing efficiencies of 83 up to 100 % (Supplementary Table S2) all formulations can be regarded as efficiently self-healing [66]. This is in line with findings that shear-thinning as well as self-healing properties are mainly associated with the reversibility and dynamic nature of physical forces between polymer chains [67], which in our case are primarily hydrophobic interactions and hydrogen bonds. Only if in the following procedure non-reversible chemical bonds are introduced by photo-crosslinking of the functional MA groups, the before dominating dynamic nature of interaction forces and thus the self-healing properties of the hydrogel are eclipsed as the polymer network is now held together strongly by covalent bonds. However, by comparing the results obtained for HA_40_HD_15_ and HA_40_HD_15_MA_18_ it becomes evident that the MA modification influences the rheological properties of the obtained physically crosslinked hydrogels also before covalent photo-crosslinking. This observation can be explained by changes in hydrogen bonding through the modification of hydroxyl groups of HA with MA. These changes may affect intra- as well as intermolecular hydrogen bonding and interaction of the polymer with the solvent [34], which might contribute to the observed rheological differences.

Photo-crosslinking of 4 %(w/w) HA_40_HD_15_MA_18_ and HA_80_DD_15_MA_13_ reveal storage moduli, which increase from 2313 Pa to 2398 Pa, respectively, after 2 min UV irradiation up to 5980 Pa and 7043 Pa after 10 min irradiation (Fig. 3(d)). The photo-crosslinking kinetics of 4 %(w/w) HA_80_DD_15_MA_13_ were further rheologically studied in dependence of the irradiation time, which was set as 2 min, 5 min or 10 min (Fig. 3(e)). In the beginning of the measurement, all samples show a curing lag time of approximately 2 min. This indicates either the absence of any crosslinking process during this period or, more reasonably, a slow initiation phase of the reaction, during which changes in mechanical properties are too subtle to be detected by the rheometer. The maximally reached storage modulus increased with longer irradiation times. In contrast to photo-curing gelatin methacrylate (GelMA) hydrogels [29], the crosslinking reaction of HA_80_DD_15_MA_13_ hydrogels stopped immediately upon cessation of UV light exposure. Increasing the concentration of the utilized photoinitiator Irgacure 2959 from 0.1 %(w/w) to 0.4 %(w/w), a significant acceleration of the polymerization speed without initial curing lag time could be observed, visible as a rapid increase of the storage modulus. In contrast to the other samples, the storage modulus of this hydrogel reached its maximum value, which additionally exceeded those of the other hydrogels, before the UV irradiation was ceased, i.e. within the first 5 min. Moreover, a slight decrease of the storage modulus can be noticed in this curve, which could be related to volume shrinkage of the measured hydrogel due to the crosslinking reaction. This in turn could reduce the contact surface with the rheometer plates. As described above, the crosslinking reaction stopped when ceasing the UV exposure. Via two additional irradiation cycles, each with 2 min of UV illumination, the possibility to reinitiate the photo-crosslinking reaction was demonstrated (Fig. 3(f)). This attribute can be utilized to adjust the mechanical properties of the final hydrogel for a specific cell hosting 3D model or to restore a hydrogel after deformation. The storage moduli can be fine-tuned between 2.3 and 7.0 kPa, which is in a physiologically relevant range with regard to natural bone marrow (2–4 kPa at physiological temperature) [19]. Estimating mesh sizes from these storage moduli (Supplementary Table S3) yields values of 8.5 to 12.3 nm for the developed hydrogels, and 10.2–12.9 nm for bone marrow, which is sufficient to allow diffusion of small metabolites or even cytokines [68]. Other hydrogels made of natural components including GelMA, different HA-derivatives, as well as compositions of HA-derivatives with GelMA or alginate, are also suitable to prepare bioinks with storage moduli in that range (Supplementary Table S4). However, the unique crosslinking combination and the crosslinking dynamics, which allow to fine-tune the mechanical properties of the hydrogel via the length of irradiation, discriminate the current innovation from previous approaches. Conclusively, the rheological properties of the developed hydrogels make them interesting for bioprinting of bone marrow analogues due to their shear-thinning, self-healing and bone marrow-mimetic behavior.

### 3.3 3D printability of methacrylamidated hydrophobically modified HA-based hydrogels

**Fig. 4:**
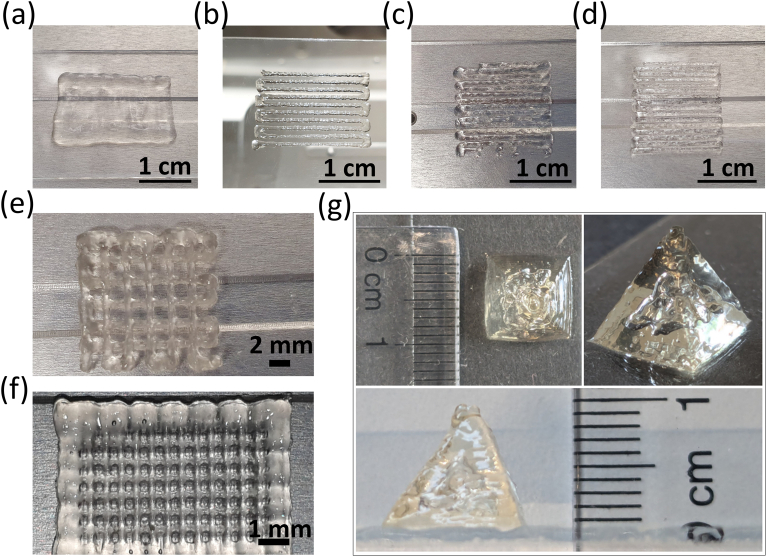
3D printing of HA-based hydrogels. Images show the 3D printability of HA_40_HD_15_MA_18_ hydrogels at polymer concentrations of (a) 3 %(w/w), (b) 4 %(w/w), (c) 8 %(w/w) with 0.450 MPa printhead air pressure and (d) 8 %(w/w) with 0.722 MPa printhead air pressure. (e, f) Representative images are shown of 3D-printed multilayer constructs using a 4 %(w/w) HA_40_HD_15_MA_18_ hydrogel with (e) 2 mm inter-filament distance and (f) 0.5 mm inter-filament distance. (g) Images of a printed pyramid using 4 % (w/w) HA_80_DD_15_MA_13_ printed in 46 layers, shown from the top (top-left image), and from the side (bottom image), next to a ruler indicating the dimensions of 1 cm base side length and 1 cm height.

To initially optimize the 3D printing of the hydrophobically and MA-modified HA, HA_40_HD_15_MA_18_ hydrogels with different polymer concentrations were tested for extrusion-based 3D printing. For this purpose, hydrogels with 3 %(w/w), 4 %(w/w) and 8 %(w/w) were prepared and 3D-printed into a single-layered construct with parallel lines (Fig. 4(a–d)). On the one hand, the printed filament completely collapses, if the polymer concentration is decreased to 3 %(w/w) and the printhead air pressure and/or printing nozzle diameter is not adjusted (Fig. 4(a)). On the other hand, increasing the concentration to 8 %(w/w) leads to needle clogging, which results in interrupted extruded filaments (Fig. 4(c)). This can partly be compensated by drastically increasing the printhead air pressure (Fig. 4(d)). However, at such high air pressures, it is hard to balance a smooth and steady material flow without overdosing versus easily occurring needle clogging.

As this initial printability assessment revealed the best results for hydrogels with a concentration of 4 %(w/w), this concentration was chosen for further semi-quantitative analysis of the printability by determining the strand width, printability factor and uniformity (Fig. S6) of extruded grid-like structures. The strand width varies from 547 to 849 μm upon extrusion from a nozzle with an inner diameter of 330 μm (Fig. S6(b)). These values are in the same range as also observed for other 3D-printed HA-based hydrogels [42] and indicate spreading of the printed filament upon deposition on the surface. The strand width appears to be lower for hydrophobically modified HA_40_ than HA_80_ hydrogels, however, this effect is not observed in the MA-modified HA-based hydrogels. The printability factor (Pr) is between 0.86 and 0.91 for all printed hydrogels, irrespective of the molecular weight of the base polymer HA, the length of the alkyl side chains or the presence of MA groups (Fig. S6(c)). A Pr < 1 indicates that the pores are rounded due to filament merging at crossing points [69], which is an expected result due to the rheological properties of the developed hydrogels. Moreover, the uniformity assessment of the strands in the printed grid-like structures reveals values between 0.81 and 0.95 (Fig. S6(d)). These values are reasonable in light of the observed filament merging at the crossing points, which leads to curved outlines of the filaments within the grid (Fig. S6(a)).

The 4 %(w/w) MA-modified hydrogel allows a continuous extrusion with a well-defined filament (Fig. 4(b), Fig. S7) and was further used for the printing of multi-layered square- or rectangle-shaped grid-structures (Fig. 4(e–f), Fig. S8). Although only at the edges, the construct with an inter-filament distance of 0.5 mm displays some signs of coalescence (Fig. 4(f)), whereas the construct with an inter-filament distance of 2 mm shows higher shape fidelity without any signs of coalescence (Fig. 4(e)). Flow and coalescence of neighboring filaments is prevented by a fast healing of the extruded filaments, which originates in the rapid reassembly of the dynamic physical crosslinks, being mainly caused by hydrophobic interactions between the small alkyl chains. To further enhance the filament circularity and hence the shape fidelity, a photo-crosslinking step can be applied after each printed layer [30]. A final curing step can be carried out in order to increase the stability of a printed construct, especially for cell culture applications. Printing pyramids with a base side length and height of 1 cm demonstrates the stability of the printed constructs and the self-standing ability of the developed ink (Fig. 4(g)).

HA has a strong water binding capacity. Therefore, the swelling behavior of printed HA_40_HD_15_MA_18_ and HA_80_DD_15_MA_13_ hydrogels was analyzed (Fig. S9). HA_80_DD_15_MA_13_ hydrogels show larger swelling than HA_40_HD_15_MA_18_ hydrogels and reach their equilibrium after 30 min, while HA_40_HD_15_MA_18_ swell less and reach their equilibrium already after 10 min. These results suggest higher crosslinking degrees in the HA_40_HD_15_MA_18_ hydrogels than the HA_80_DD_15_MA_13_, as higher crosslinking is known to be associated with lower swelling [70].

### 3.4 3D bioprinting of HA_40_HD_15_MA_18_ and HA_80_DD_15_MA_13_ hydrogels

**Fig. 5:**
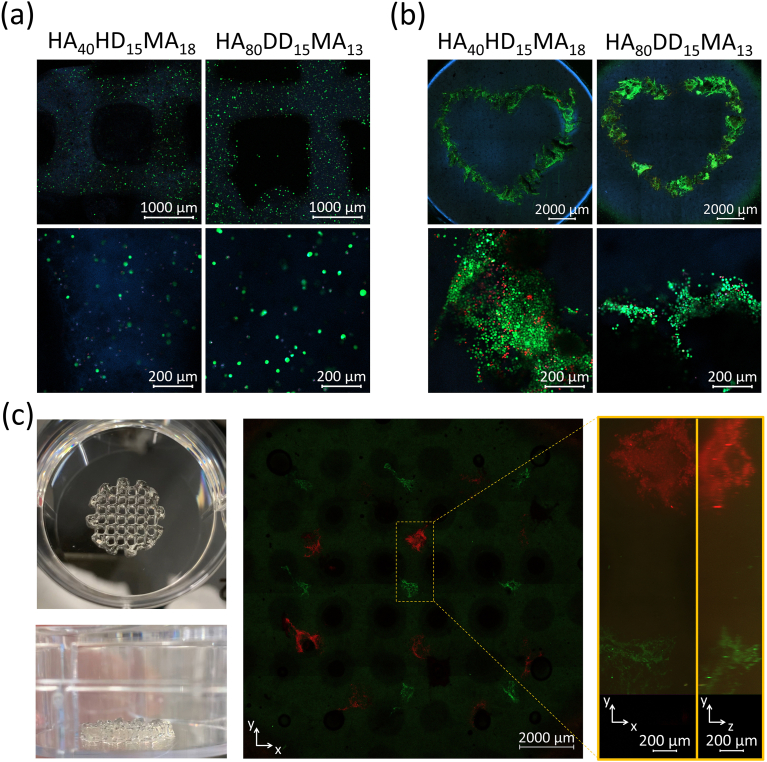
**3D bioprinting of HA_40_HD_15_MA_18_ and HA_80_DD_15_MA_13_ hydrogels.** (a) 2 %(w/w) HA-based hydrogels including iMSC#3 cells (1x10^7^ cells mL^−1^) were 3D-bioprinted into a grid structure with 10 layers. (b) 4 %(w/w) HA-based hydrogels were printed into a filled flat cylinder consisting of 10 layers, into which KG-1a cells (3x10^7^ cells mL^−1^) were injected in a heart shape in a second bioprinting step in 4 layers. (a, b) After 24 h the viability was analyzed via live-dead staining and images were taken with a confocal laser scanning microscope with 2.5x (top) and 10x (bottom) objectives. Living cells were stained with Calcein-AM (green), while dead cells can be seen in red (propidium iodide). All nuclei were stained with Hoechst 33342 (blue). The upper panel in (b) shows entire hydrogels recorded in *tiles and positions* mode. (c) 4 %(w/w) HA_40_HD_15_MA_18_ hydrogels were printed into a grid shape with 10 layers. In a second bioprinting step, pre-stained iMSC#3 (red) and KG-1a cells (green) with cell concentrations of 1x10^7^ cells mL^−1^ were injected into the nodes of the grid in 5 layers. Photographic images of top and side view of the printed hydrogels in a 6-well plate are depicted on the left. Confocal laser scanning microscopic images show an entire gel acquired in *tiles and positions* mode with a 2.5x objective (middle). The close-up on the right shows two neighboring nodes with assemblies of injected cells (iMSC#3 cells in red, KG-1a cells in green) in top view (x/y) and orthogonal view (y/z), captured as z-stack in combination with the *tiles and positions* mode using a 10x objective (n = 3).

HA is an essential component of the ECM, for example in the HSC niche being expressed by HSCs as well as by other niche cells like MSCs (reviewed by Ref. [8]). It was therefore assumed that the developed HA-based bioinks will show a high cytocompatibility and are well suitable for 3D bioprinting. To increase the cytocompatibility, the polymer was dissolved in cell culture media and LAP was used as photoinitiator instead of Irgacure 2959 for all bioprinting experiments. An LED-based point source with a monochromatic spectrum was used for UV illumination to limit energy input and thus potential phototoxic effects. Future use of blue light, might additionally mitigate this risk. Furthermore, the polymer concentration in the ink was reduced to 2 %(w/w), because the strong hydrophobic interactions of the HA_40_HD_15_MA_18_ and the HA_80_DD_15_MA_13_ polymers hinder successful inclusion of cells into 4 %(w/w) gels, potentially due to high shear forces occurring during mixing and bioprinting, which lead to cell rupture and low cell survival (Fig. S10). This concentration reduction aimed to decrease shear stress for the cells during mixing into the ink and to allow for lower air pressure during the bioprinting process. The adapted formulation enables bioprinting of hydrogel grid constructs including iMSC#3 cells (Fig. 2 (a)) by resuspending the cells in the prewarmed hydrogels and bioprinting with a directly dispensing printing head. During and after bioprinting, the hydrogels were chemically crosslinked with UV light and cell culture medium was added to the samples as soon as the bioprinting and crosslinking process was completed. After a growth period of 24 h, a live-dead staining was performed to analyze the cytocompatibility of the HA-based bioinks (Fig. 5(a)). Both, in the HA_40_HD_15_MA_18_ and the HA_80_DD_15_MA_13_ hydrogels many living cells are visible. Less dead cells seem to be present in the HA_80_DD_15_MA_13_ hydrogels. These results confirm the expected cytocompatibility of the HA-based hydrogels and show that the chosen 3D bioprinting parameters do not lead to exceeding shear stress on the cells so they can survive.

Due to its slightly better performance in the initial direct extrusion-bioprinting experiments, HA_80_DD_15_MA_13_ was chosen for further analysis of the suitability of the developed ink for direct bioprinting of both, stromal as well as hematopoietic cells. The cells were bioprinted and cell viability was assessed over the course of 14 days (Fig. S11). Lowest cell viabilities are observed at early timepoints. Given the biocompatibility of HA itself, this observation might be the result of shear forces acting on cells during mixing into the ink and the bioprinting process, interactions of the alkyl side chains of the hydrophobically modified HA with cell membranes or phototoxic effects. Since C_12_- and C_16_-derivatized HA developed for osteoarthritis or ophthalmic treatments proved high cytocompatibility in previous studies [71,72] and HD-functionalized HA, called HyaDD® 4 is even commercialized as Hymovis® and approved for treatment of osteoarthritis [[73], [74], [75]], the functionalization with alkyl side chains can be considered safe and not harmful for the cells. Furthermore, particularly the KG-1a cells with their poorly developed cytoskeleton, which makes them very sensitive to mechanical damage, initially show higher numbers of dead cells. Therefore, it seems likely that the shear forces during mixing of cells into the ink and the printing process are the primary reason for the observed lower cell viabilities at early time points. At all timepoints, iMSC#3 cells show higher cell viabilities than KG-1a cells, reflecting their higher resistance to mechanical stress. For both cell types, viability increases with time to 90 % for iMSC#3 and 79 % for KG-1a cells (Fig. S11(b)).

Over the culture period, we observed the appearance of cell clusters derived from initially single cell suspensions of KG-1a and iMSC#3 cells (Fig. S12). First and foremost, this observation demonstrates that the cells are able to multiply within the hydrogels. Secondly, it suggests that upon cell division the cells stay in place and form colonies similar to cell behavior observed in semi-solid media, which are commonly used for HSPC colony-forming unit assays. Thirdly, this appearance of large clusters particularly in KG-1a cell cultures shows that the cells might be able to remodel or degrade the applied HA-based hydrogels. Future studies on the degradation of the hydrogels in dependence of the applied base polymer (HA_40_ or HA_80_), their modifications, polymer concentrations and the applied cell type will help to broaden the understanding of the interaction of different cell types with HA-based biomaterials.

To assess the differentiation of the two cell types after bioprinting in the applied hydrogels, expression of PU.1 and HOXA9 was analyzed for KG-1a cells and alkaline phosphatase (ALP) and collagen type I (COL1A1) for the iMSC#3 cells (Fig. S13). PU.1 and HOXA9 play crucial roles in hematopoiesis and leukemia. Their downregulation in KG-1a cells after 7 and 14 days of culture in the bioprinted hydrogels in comparison to conventional 2D maintenance cultures is indicative of differentiation [[76], [77], [78]], which could be related to higher cell densities in the bioprinted constructs than in the maintenance culture. ALP and COL1A1 are key markers of osteogenic differentiation [79]. Both are down-regulated in iMSC#3 cells after 7 days of culture in the bioprinted hydrogels in comparison to conventional 2D maintenance culture. After 14 days, the expression levels are again comparable to the maintenance culture. These results indicate that after a deprivation phase, iMSC#3 cells are maintained in the bioprinted hydrogels similar to the conventional maintenance culture without osteogenic differentiation, as the cells were grown in maintenance media and not differentiation media.

Although grid structures could be 3D-printed with the 2 %(w/w) hydrogels, high resolutions were not possible. As the cells could not be encapsulated in the 4 %(w/w) hydrogels before bioprinting, a two-step bioprinting process was established. This approach benefits from the already described self-healing properties of the HA-based hydrogels before chemical crosslinking. In a first step the hydrogels were printed into the desired shape and in a second step, the cells suspended in cell culture medium were injected directly into the already printed hydrogels (Fig. 2 (b)). Via dynamic hydrophobic interactions and hydrogen bonds between the alkyl-modified HA polymers, injuries in the printed hydrogel, caused by the printing needle, are reversible [43,59] and lead to cell encapsulation during resealing. The subsequent photo-initiated chemical crosslinking therefore results in intact HA-based cell-hosting hydrogels (Fig. 5(b–c), supplementary video 1).

As a higher polymer concentration could potentially be more cytotoxic than the already tested 2 %(w/w) gels, the cell viability of iMSC#3 (Fig. S14) and KG-1a cells (Fig. 5(b)) was examined via live-dead staining. For these tests, the cells were injected in a heart shape into a compact flat hydrogel cylinder. Many living cells can be observed, both, in the HA_40_HD_15_MA_18_ and the HA_80_DD_15_MA_13_ hydrogels. Again, less dead cells appear to be present in the HA_80_DD_15_MA_13_ hydrogels. It can be concluded that also the 4 %(w/w) HA-based hydrogels possess a high cytocompatibility and are well suitable for 3D cell culture models. This has been expected as several studies using HA-based or HA-containing bioinks for 3D-bioprinting in diverse tissue engineering applications demonstrated high levels of cell viability [[40], [41], [42],[80], [81], [82], [83], [84], [85]] (Table S4).

Injecting cells during a second bioprinting step into the already printed hydrogel is advantageous, because the experimental handling is easier and quicker if cells are resuspended in culture medium instead of being mixed into the hydrogel. This in turn leads to less shear stress for the cells. The shear stress is further reduced during the bioprinting process itself, as significantly less pressure needs to be applied to print cells suspended in low-viscosity solutions such as cell culture medium [81].

As outlined above, the HA-based hydrogels presented here were primarily developed as bone marrow-mimetic matrices intended for application in 3D *in vitro* models of the HSC niche. Besides HSCs, the HSC niche homes a variety of other cell types being essential for the self-renewal and controlled differentiation of HSCs [[3], [4], [5]]. Bone cells like osteoblasts and especially MSCs as their progenitors are of particular interest in this context [3,4,86,87]. Therefore, we tested the possibility to include and precisely place iMSC#3 cells as model for MSCs and KG-1a cells as model for HSCs into the same hydrogel using the established two-step bioprinting procedure. Stromal cells (such as iMSC#3) and hematopoietic cells (such as KG-1a) strongly differ in their requirements concerning culture media (rich versus minimal media, serum content), growth mode (adherent versus suspension culture) and bioprinting. While stromal cells develop a robust actin cytoskeleton, hematopoietic cells as suspension cells possess a comparatively loose cortical actin meshwork resulting in lower cytoskeletal integrity [88]. At the same time, stromal cells are larger than hematopoietic cells (diameters of 15–30 μm versus 4–12 μm, respectively [89]). Therefore, finding a balanced ink and integrated printing processes that work for both cell types (small, compliant hematopoietic versus larger and stiffer stromal cells) and allow their inclusion into a bioprinted construct in a single process is challenging. To evaluate the applicability of the two-step bioprinting procedure for this purpose and for more delicate hydrogel structures, a HA_40_HD_15_MA_18_ hydrogel was initially printed into a grid shape. Subsequently, the two different cell types (KG-1a and iMSC#3) were injected into the nodes of the grid in an alternating manner (Fig. 2(c and d)). The cells were pre-stained with two different cell-tracking dyes before bioprinting, so they could be distinguished during microscopy (Fig. 5(c) for HA_40_HD_15_MA_18_ and Fig. S15 for HA_80_DD_15_MA_13_). It can be observed that the cell populations are precisely placed into the hydrogel in all three dimensions while the grid structure of the gel itself stayed intact although the external nozzle diameter was nearly as thick as the hydrogel strand diameter. The cells are found as assemblies of hundreds of cells in different levels in z-direction indicating that the cells are integrated into the hydrogel structure and not flowing out of the gel. The different cell types are present separately from each other and do not mix.

As mentioned above, many studies have used a HA-containing or HA-based bioink for 3D bioprinting. However, if high resolution and shape fidelity were reached, the bioinks mostly either contained additives, as for example gelatin or alginate to increase the viscosity of the ink [82,83], were mechanistically relying on guest-host interactions and therefore required synthesis of at least two different polymers [84], or required supportive matrices such as support layers in embedded bioprinting [80]. Only a few studies have reported single-component HA-based inks for extrusion-based bioprinting [31,[40], [41], [42]]. In comparison to multi-component inks, these excel by simplifying the bioink formulation and processing. In addition, the chosen dual crosslinking strategy of the here presented bioink eliminates the need for pre-printing gelation steps, thereby further facilitating the process. Moreover, using low molecular weight HA (40 and 80 kDa) facilitates the handling and thus enhances the robustness of the synthesis route in comparison to high molecular weight HA as the latter yields highly viscous solutions [43]. The bioprinting of constructs with large macropores (as shown in Fig. 5(c)), yields structures which combine printed macropores with hydrogel-inherent nanoporosity (Table S3) and, therefore, allow diffusion of nutrients, metabolites and cytokines. Most importantly, to the best of our knowledge, this is the first report of a bioink and approach, which allow bioprinting of stromal and hematopoietic cells in one bioink and one integrated process. Thus, we provide a first proof-of-principle demonstrating that it is possible to simultaneously bioprint these two fundamentally different cell types, which are crucial for the bone marrow in an ink mimicking the chemical (composition based on HA) and biophysical properties (rheological behavior, mesh size) of the bone marrow. This paves the way for future bioprinting of primary human HSPCs and supporting niche cells such as MSCs to create bone marrow models suitable for applications such as disease modeling, personalized drug screening, or targeted multiplication or differentiation of HSPCs for cellular therapies.

Beyond applications in bioengineering bone marrow models, the established two-step 3D bioprinting procedure is generally advantageous if different cell types shall be included into a 3D construct. In an easily adaptable way, different cell types can – precisely in all dimensions – be placed into pre-printed hydrogel structures. The results are 3D-printed HA-based hydrogels with excellent shape fidelity, additionally hosting viable cells of different kinds in accurately defined spaces.

## 4 Conclusions

In conclusion, this study introduces a bioink formulation based on dual-functionalized HA designed to emulate the intricate 3D microenvironments of the bone marrow. Our approach successfully addresses the challenges associated with physiologically relevant placement of various cell types in bone marrow-mimetic matrices, which is crucial for functionality of the native and thus also artificial niches. By leveraging both, alkyl side chains for enhanced physical crosslinking and methacrylamide groups for covalent photo-crosslinking, we have synthesized a versatile bioink demonstrating desirable shear-thinning and self-healing properties, pivotal for extrusion-based bioprinting.

The developed bioink allows for two distinct bioprinting strategies: direct encapsulation of cells into the hydrogel matrix or precise injection of cells into pre-printed constructs. Both methodologies exhibit excellent cell viability, thus broadening the potential applications of our bioink in soft tissue engineering. Critically, our bioink offers a streamlined process including a one-pot polymer synthesis strategy and bioprinting without the necessity for additional additives. The usage of HA as a natural component of the bone marrow ECM and tuning the mechanical characteristics of the ink in a range resembling natural bone marrow tissue, enhances the biomimetic quality of the printed constructs and ensures compatibility with hematopoietic and stromal cells. To the best of our knowledge, this is the first study presenting a solution to bioprint both, hematopoietic and stromal cells, within one bioink and one integrated process.

In summary, our bioink presents a compelling platform for bioprinting advanced bone marrow models and other soft tissue constructs. It is poised to facilitate future fundamental research and potential therapeutic applications by providing a robust and physiologically relevant environment for studying healthy and pathological hematopoietic processes *in vitro*.

## CRediT authorship contribution statement

**Toufik Naolou:** Writing – review & editing, Writing – original draft, Visualization, Methodology, Investigation, Formal analysis. **Nadine Schadzek:** Writing – review & editing, Writing – original draft, Visualization, Methodology, Investigation, Formal analysis. **Jonas Nolte:** Writing – review & editing, Investigation, Visualization, Formal analysis. **Susanna Spindler:** Writing – review & editing, Methodology, Investigation. **Franziska Lötz:** Investigation. **Lena Fraedrich:** Resources, Investigation. **Gerald Dräger:** Resources. **Tomasz Jüngst:** Writing – review & editing, Conceptualization. **Jürgen Groll:** Writing – review & editing, Conceptualization. **Cornelia Lee-Thedieck:** Writing – review & editing, Writing – original draft, Supervision, Project administration, Funding acquisition, Conceptualization.

## Funding

This project has received funding from the European Research Council (ERC) under the European Union's Horizon 2020 research and innovation programme (grant agreement No 757490). This work was also supported by the 10.13039/100011937Ministry of Science and Culture (MWK) of Lower Saxony, Germany through the SMART BIOTECS alliance between the Technische Universität Braunschweig and the Leibniz Universität Hannover. The project was supported in the framework of the collaborative research project Matrix Evolution, funded by zukunft.niedersachsen, a funding programme of the Lower Saxony Ministry of Science and Culture (MWK) and the 10.13039/501100001663Volkswagen Foundation.

The confocal laser scanning microscope (LSM 980, Carl Zeiss, Oberkochen, Germany) and the bioprinter (3D DiscoveryTM Evolution, RegenHU, Villaz-Saint-Pierre, Switzerland) were funded by the Deutsche Forschungsgemeinschaft (DFG, German Research Foundation) – project numbers 420505864 and 420457607.

## Declaration of competing interest

The authors declare that they have no known competing financial interests or personal relationships that could have appeared to influence the work reported in this paper.

## Data Availability

Data will be made available on request.
