# Supplementary material for: Additive-free hyaluronic acid-based bioink for 3D bioprinting of bone marrow microenvironments
Source: Mater Today Bio. 2025 Nov 5;35:102512. doi: 10.1016/j.mtbio.2025.102512 (PMC12657760; doi:10.1016/j.mtbio.2025.102512)
Supplement: Multimedia component 1 [file mmc1.pdf]

Supplementary Information

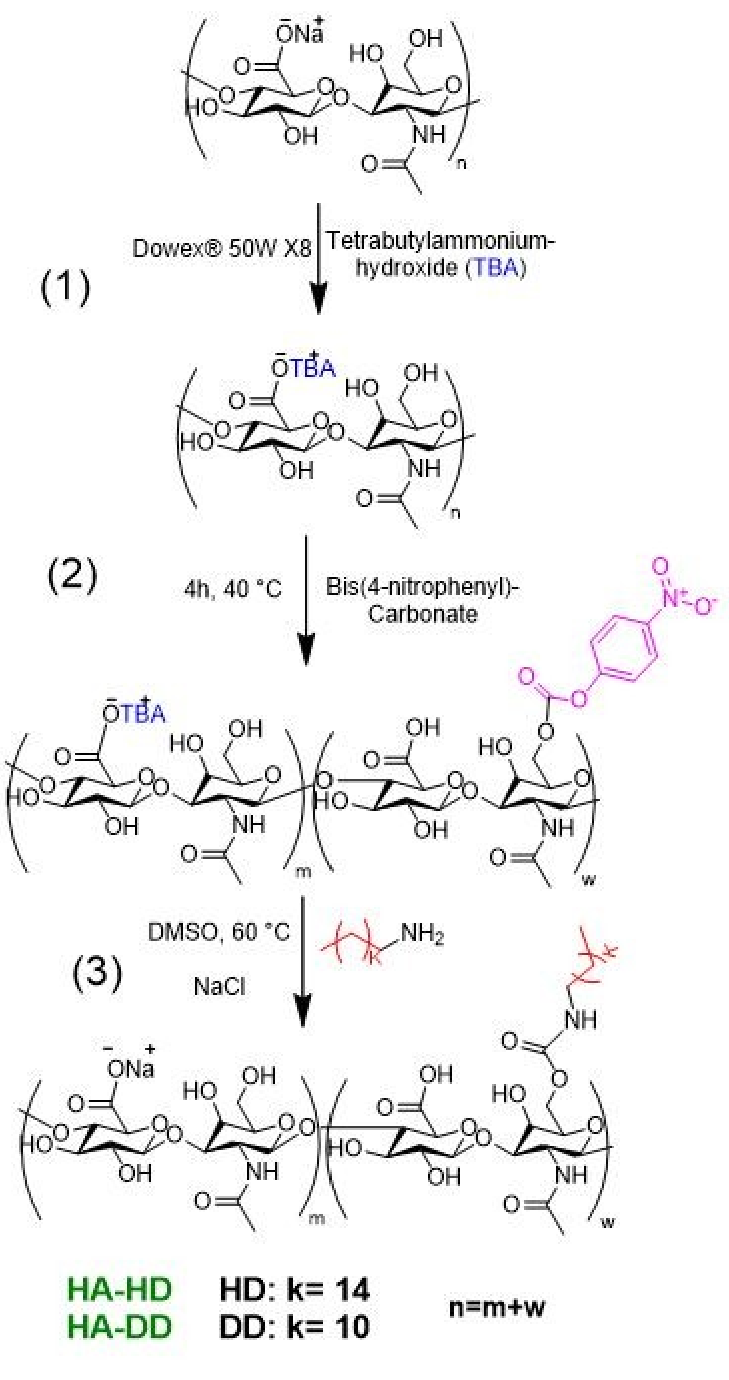

Figure S1. Synthetic pathway to prepare HA-HD and HA-DD using a three-step synthesis strategy.

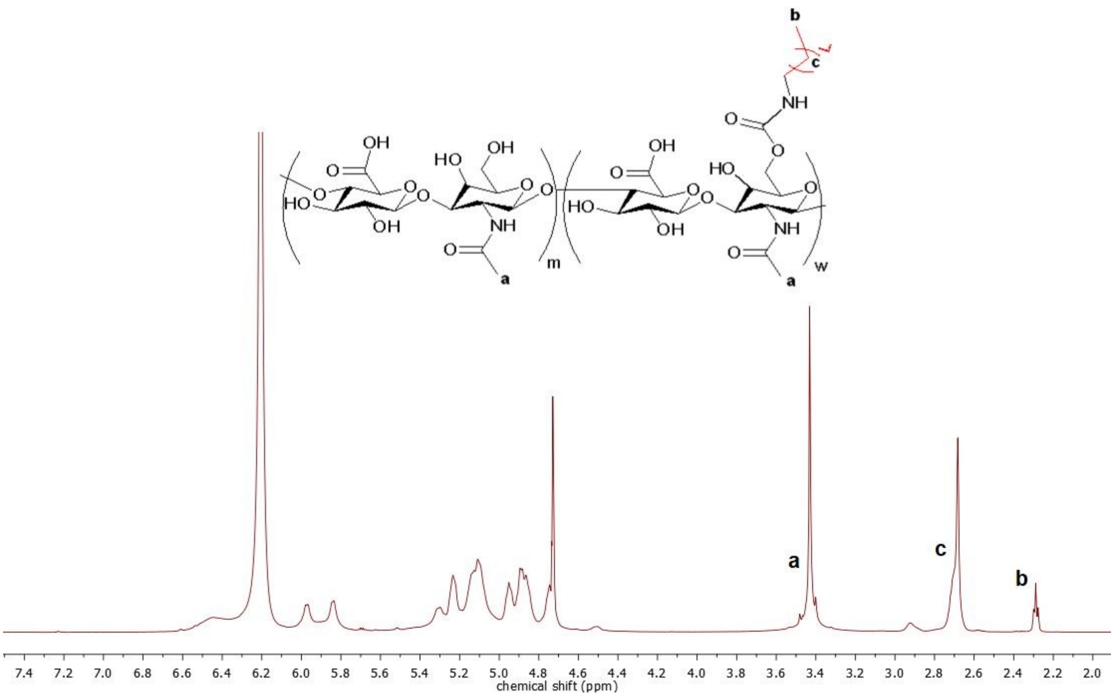

Figure S2. Representative ^{1}HNMR spectrum of HA-HD or HA-DD in D_2O/CD_3OD (70:30 %(v/v).

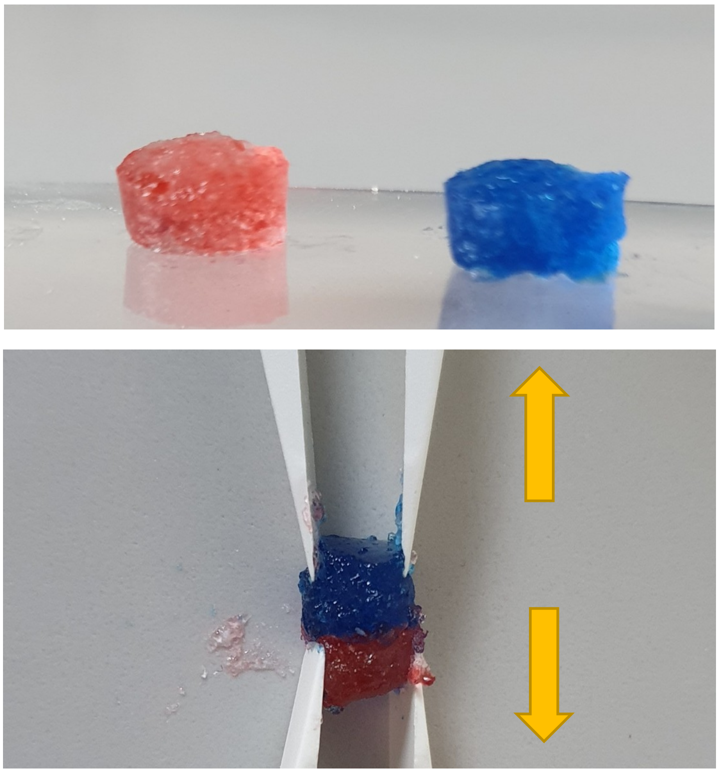

Figure S3. A red and a blue colored 6 %(w/w) $HA_{80}DD_{15}MA_{13}$ hydrogel were prepared separately (top). After placing the hydrogels on top of each other for 10 min to enable a connection of the interfaces and

10 min UV photo-polymerization afterwards, the hydrogel resists external pulling forces (bottom), showing the self-healing properties of the uncured hydrogel.

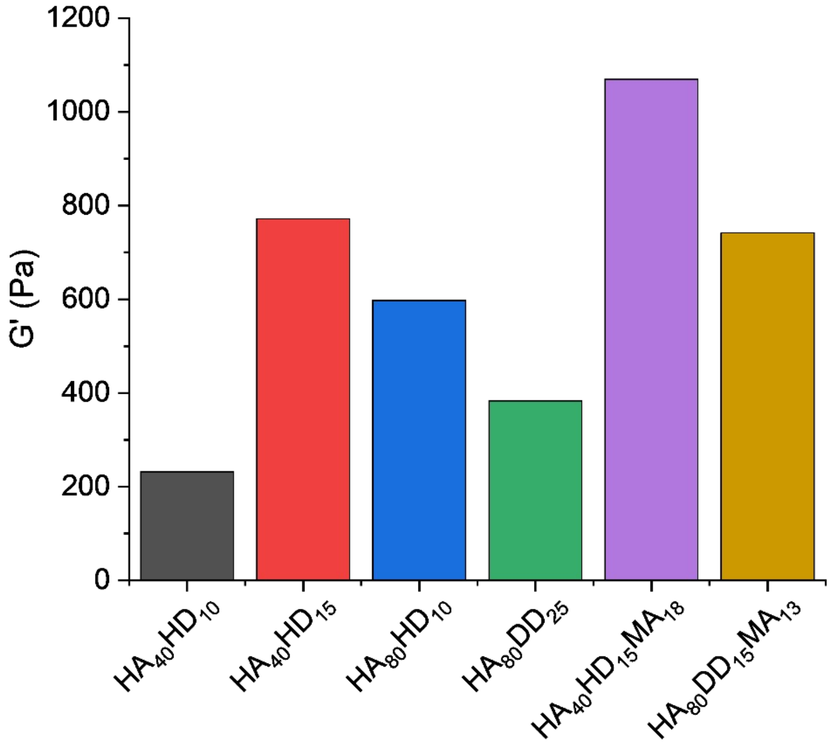

Figure S4. Storage moduli (G') of physically crosslinked 4 %(w/w) hydrogels prepared from the different HA derivatives at 1 % strain.

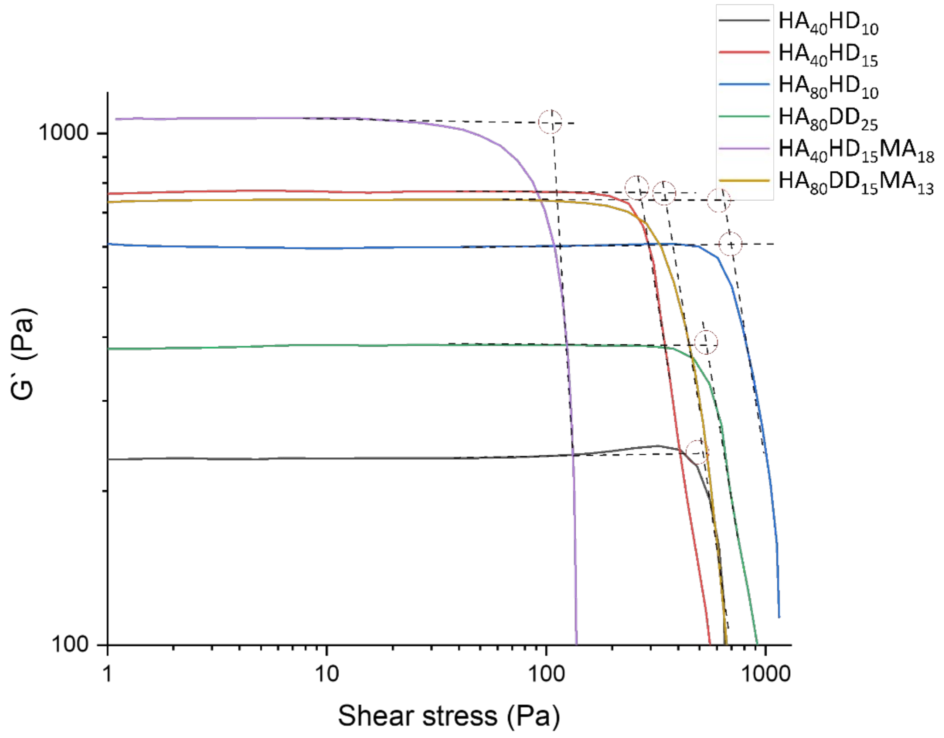

Figure S5. The storage moduli (G') of 4 %(w/w) hydrogels, prepared from hydrophobically modified

HA, are plotted as a function of shear stress. The dashed lines indicate the linear tangents identifying the yield stress in the intersection point (red circles).

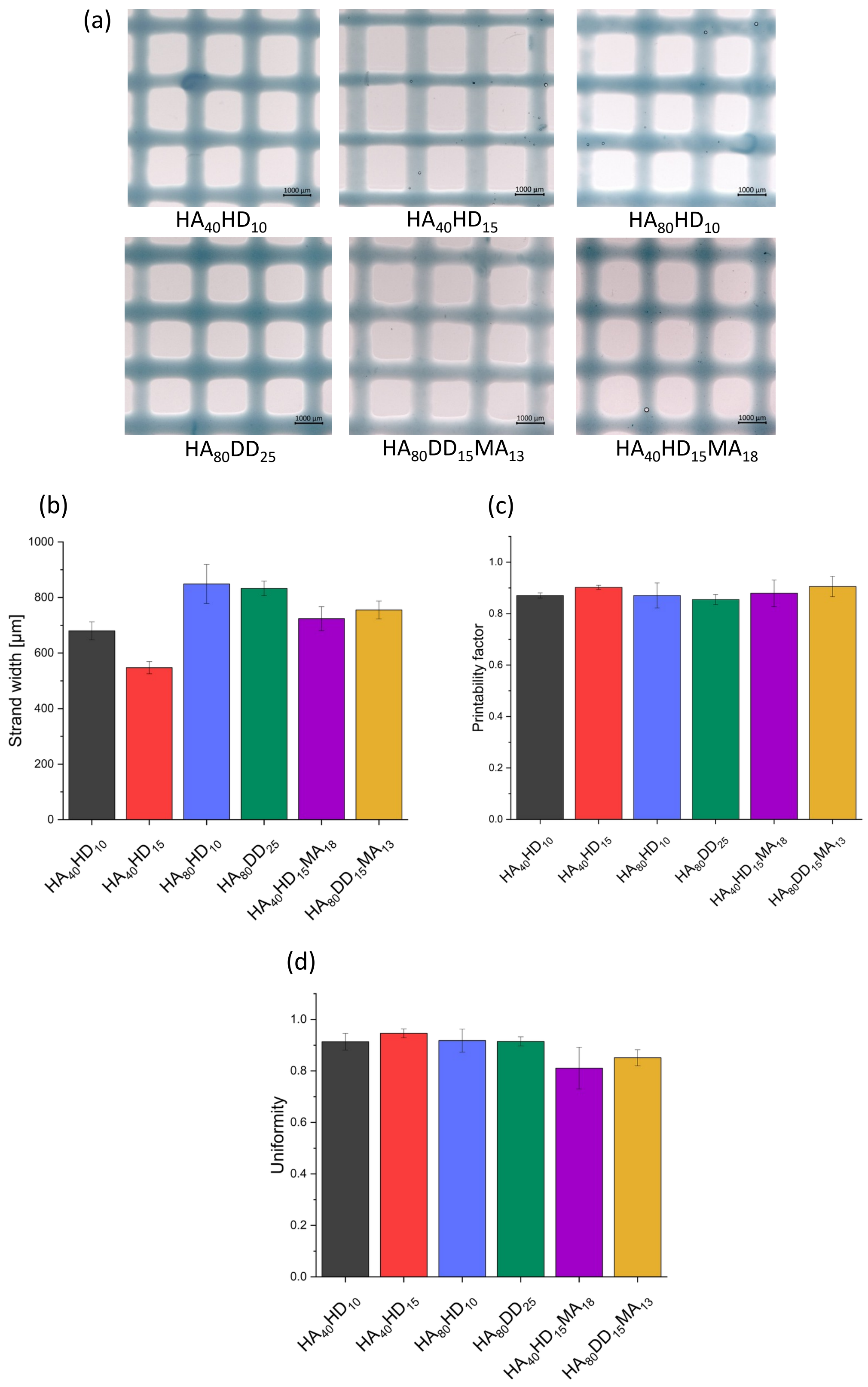

Figure S6. Printability assessment of hydrophobically modified HA-based hydrogels. Grid structures consisting of a horizontal and a vertical layer with an inter-filament distance of 2 mm were printed on a

microscopy slide and subsequently imaged with a stereomicroscope (Zeiss). Representative images of the analyzed structures are displayed in (a). Strand width (b), printability factor (c), and uniformity (d) were determined for each gel type and are shown as means with standard deviation (n=3).

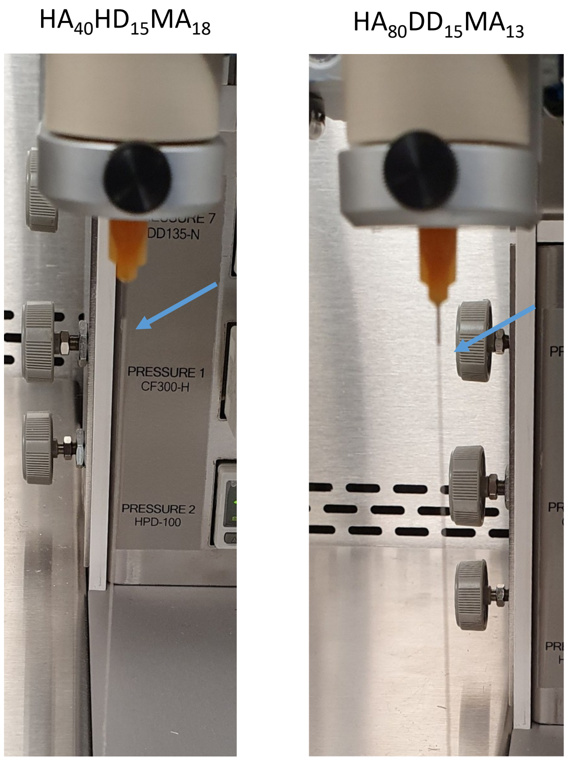

Figure S7. Filaments of 4 %(w/w) $HA_{40}HD_{15}MA_{18}$ and $HA_{80}DD_{15}MA_{13}$ hydrogels upon extrusion from the nozzle. The transparent filaments are marked with blue arrows.

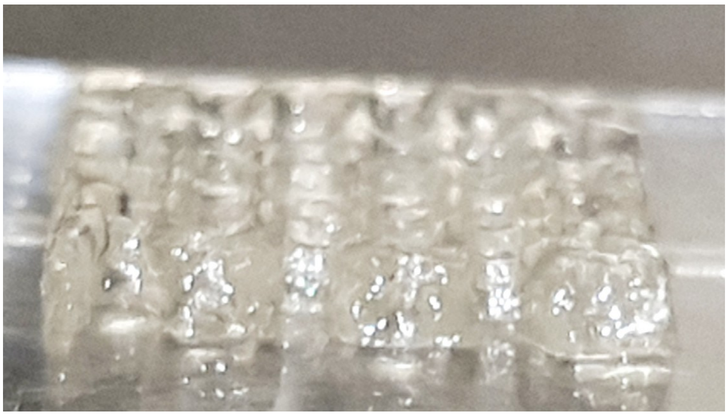

Figure S8. Side view of multilayer constructs using a 4 %(w/w) $HA_{40}HD_{15}MA_{18}$ hydrogel shown in Figure 4 (e).

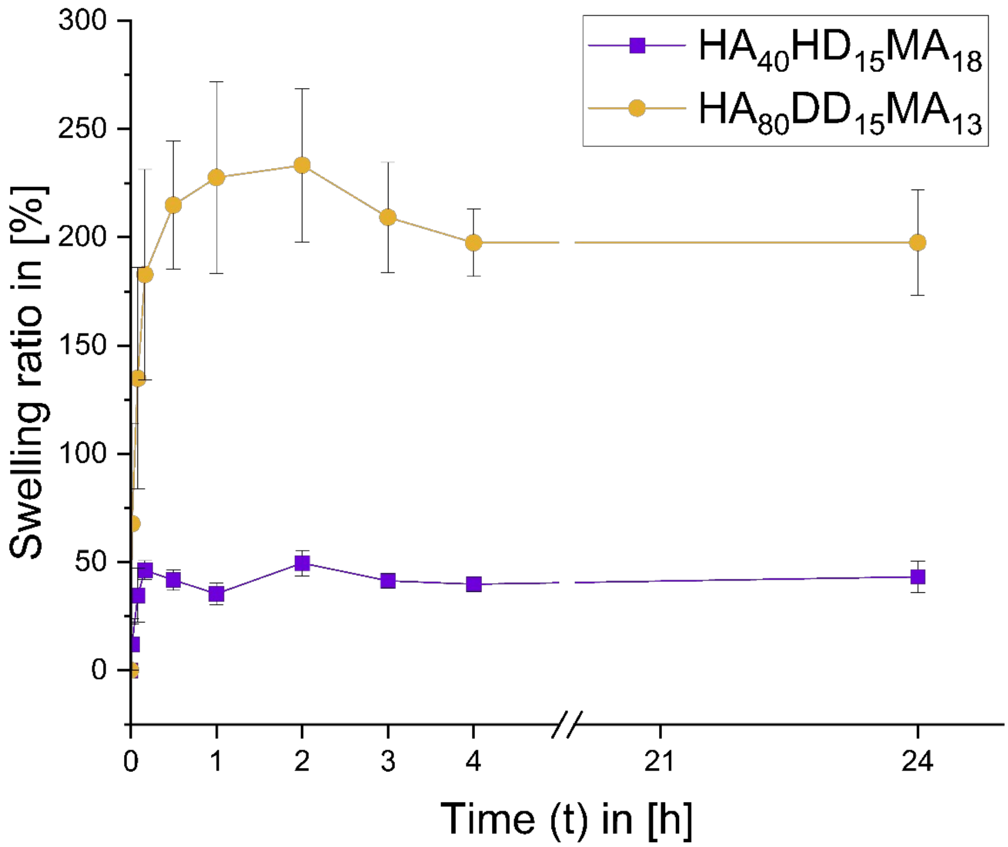

Figure S9. Swelling ratio of 4 %(w/w) $HA_{40}HD_{15}MA_{18}$ (violet) and $HA_{80}DD_{15}MA_{13}$ (yellow) hydrogels over 24 h in phosphate buffered saline. The diagram shows mean values with standard deviations from n = 6 hydrogels per polymer.

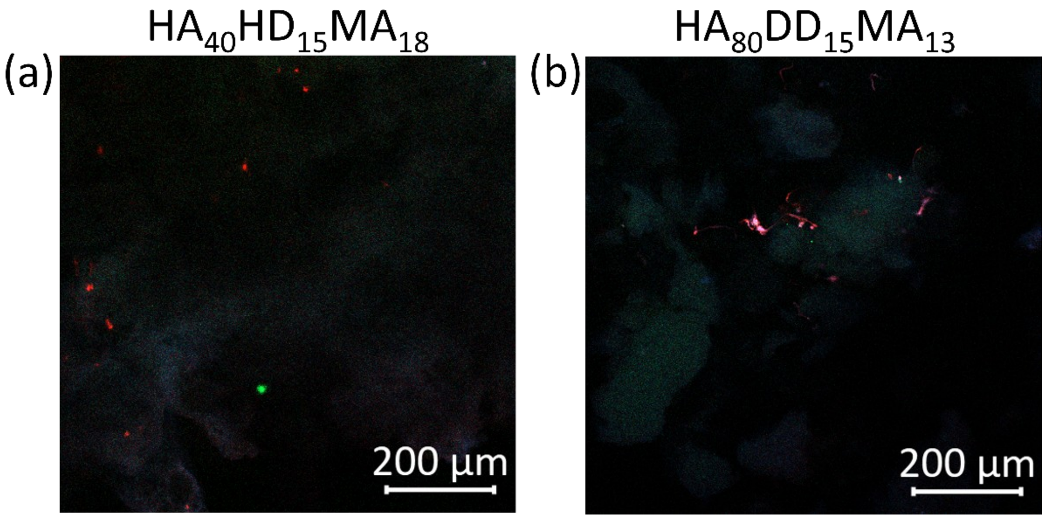

Figure S10. iMSC#3 cells ($1x10^7$ cells mL^{-1}) were mixed into HA-based hydrogels consisting of 4 %(w/v) $HA_{40}HD_{15}MA_{18}$ (a) or $HA_{80}DD_{15}MA_{13}$ (b). Subsequently, the cell-containing hydrogels were 3D-bioprinted and photo-crosslinked with blue light. After 24 h in culture, a live-dead staining with Calcein-AM (living cells, green) and propidium iodide (dead cells, red) was performed. Nuclei were stained with Hoechst 33342 (blue). Microscopic images were acquired with a confocal laser scanning microscope using a 10x objective.

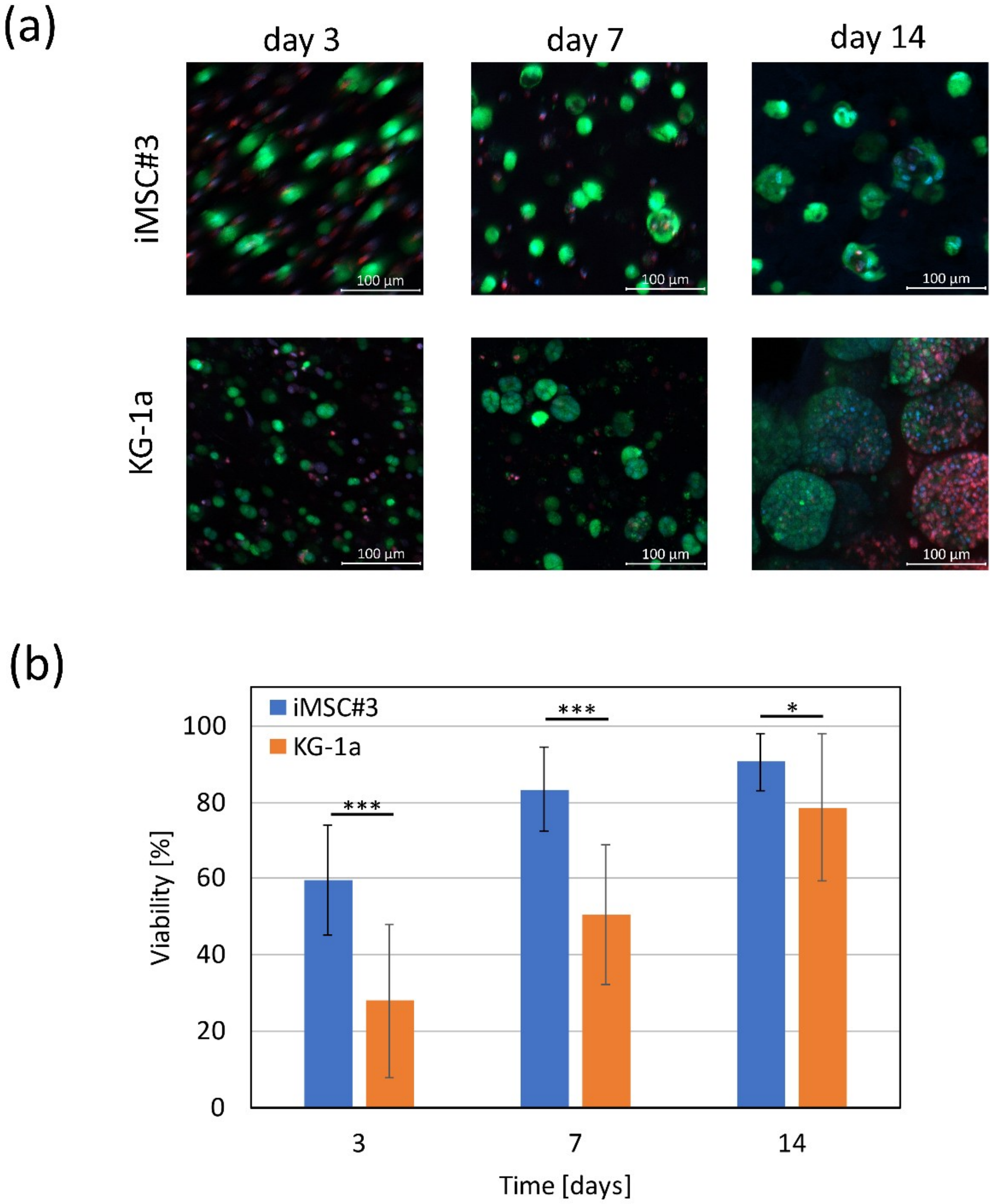

Figure S11. iMSC#3 and KG-1a cells were directly bioprinted with 2 %(w/w) $HA_{80}DD_{15}MA_{13}$ hydrogels ($5x10^6$ iMSC#3 cells mL^{-1} and $1x10^7$ KG-1a mL^{-1}). After 3, 7 and 14 days, cells were stained with Calcein-AM (green, living cells), propidium iodide (red, dead cells) and Hoechst 33342 (blue, all nuclei). (a) Representative confocal z-stack images, taken with a 10x objective, are shown as maximum intensity projections for each cell line and time point. (b) For each time point and cell line, the percentage of living cells was quantified and is depicted as mean +/- standard deviation for iMSC#3 in blue and KG-1a cells in orange. n=2 independent experiments performed in triplicates. At least 6 images from 3 replicate gels were quantified per time point and cell line. Statistical significance was tested via t-test for independent samples with unequal variance (Welch's t-test). *** indicates $p < 0.001$, * indicates $p < 0.05$.

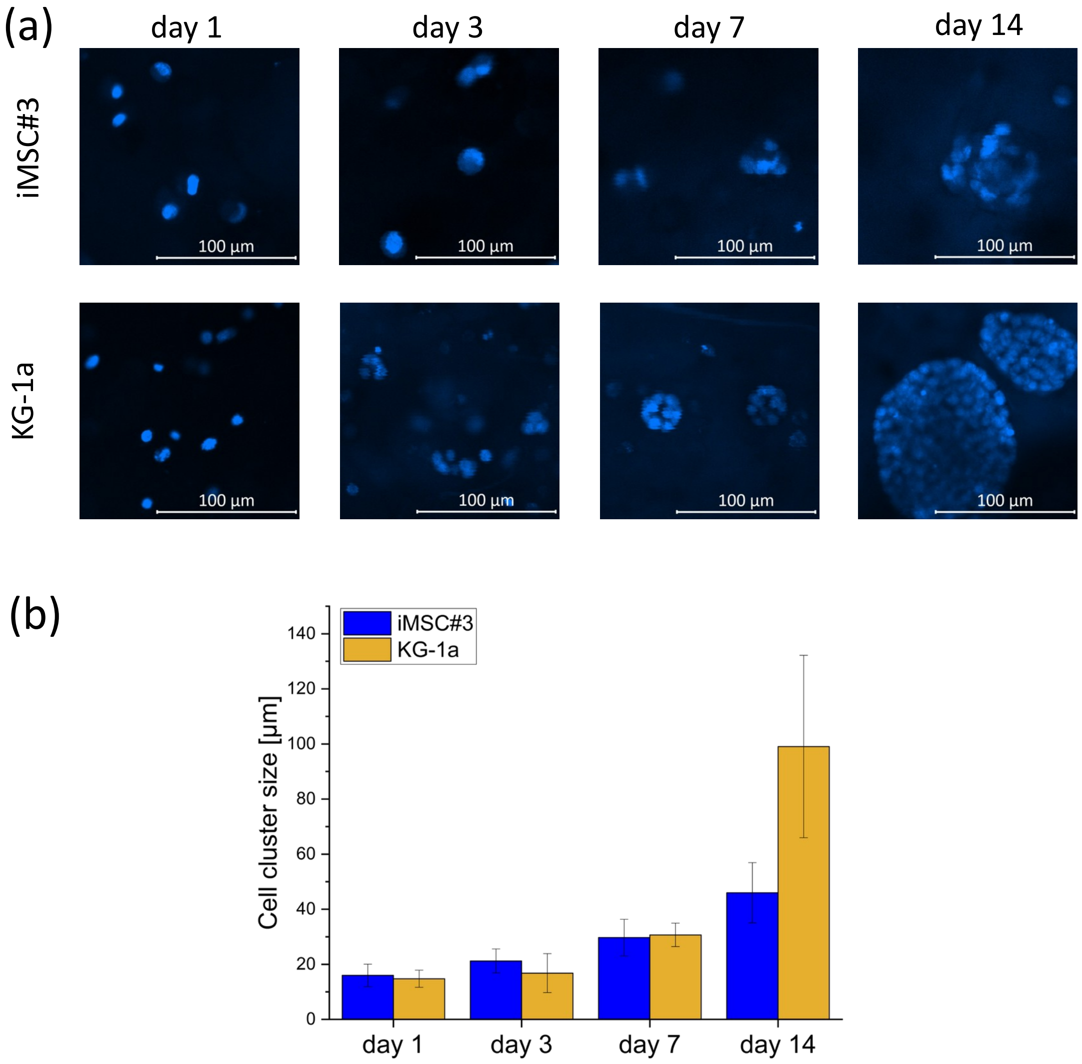

Figure S12. KG-1a and iMSC#3 cells grow to clusters upon bioprinting with 2 %(w/w) $HA_{80}DD_{15}MA_{13}$ hydrogels ($5x10^{6}$ iMSC#3 cells mL^{-1} and $1x10^{7}$ KG-1a mL^{-1}). (a) Representative confocal images of Hoechst-stained nuclei are shown for day 1, 3, 7 and 14 post bioprinting. Images were taken with a 10x objective. (b) Cluster sizes were estimated by measuring the diameters of 12 clusters from 3 different gels at each timepoint. Mean values with standard deviations are shown for KG-1a cells in orange and for iMSC#3 cells in blue. At initial timepoints (day 1 and day 3), cells are mostly observed as single cells or clusters of only a few cells. Thereafter, cells start to grow in clusters, which increase in size and cell number up to day 14.

(a)

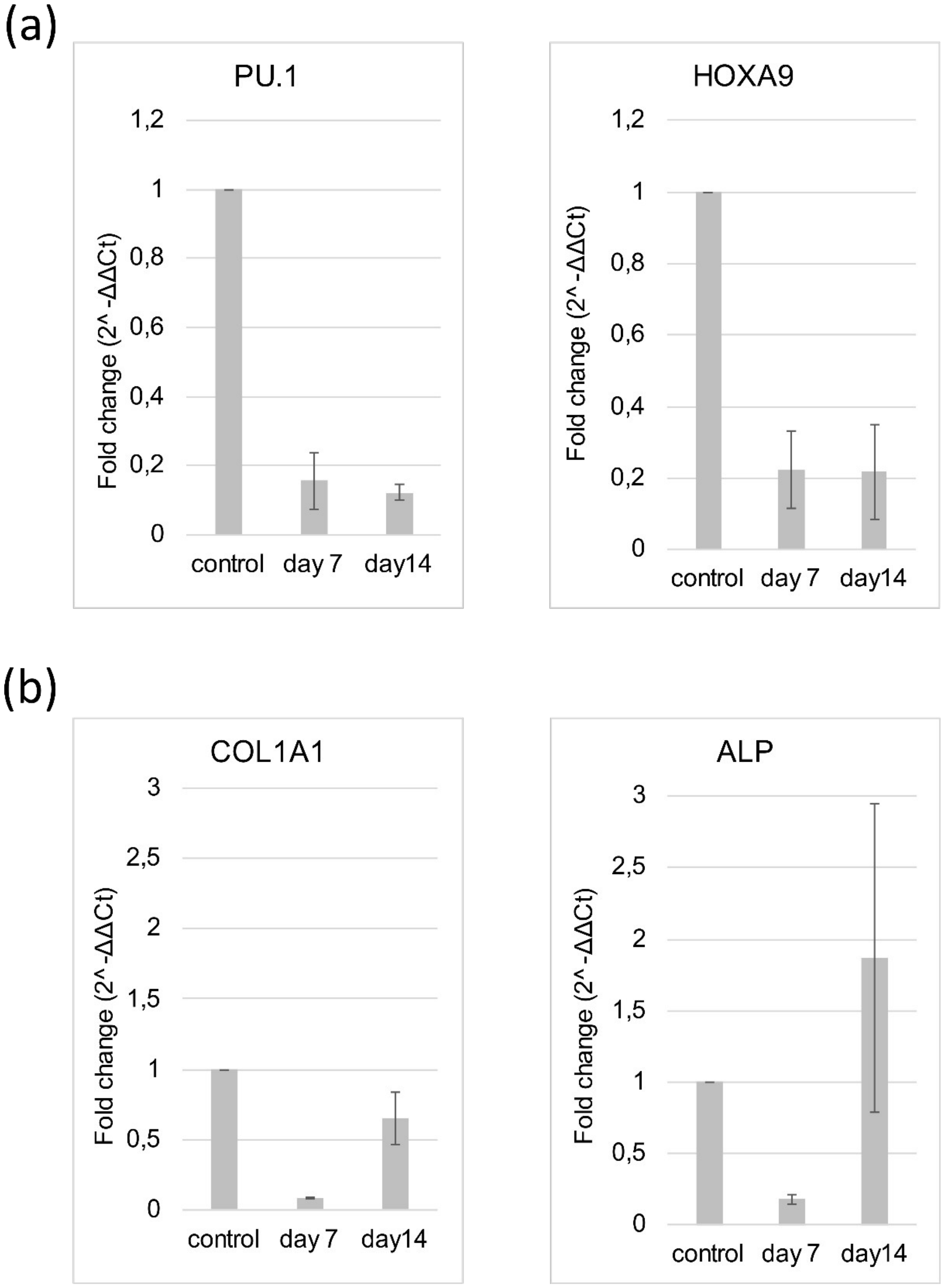

Figures S13. Differentiation of bioprinted iMSC#3 and KG-1a cells in 2 %(w/w) $HA_{80}DD_{15}MA_{13}$ hydrogels. Expression of PU.1 and HOXA9 was assessed for KG-1a cells and alkaline phosphatase (ALP) and collagen type I (COL1A1) for iMSC#3 cells and compared to the expression in conventional 2D maintenance culture. Means of fold changes are displayed with the standard deviations (n=2 independent experiments, analysis in triplicates).

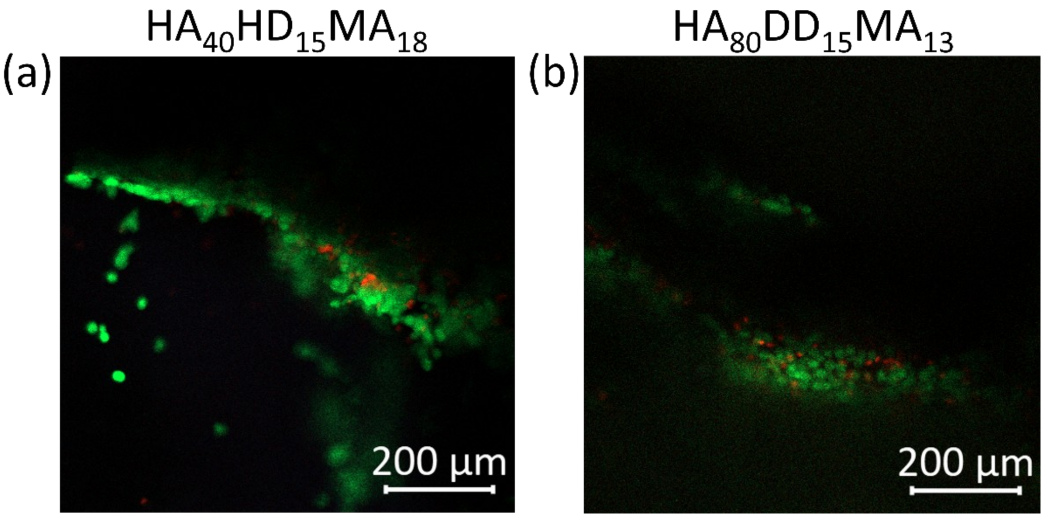

Figure S14. Hydrogels prepared from 4 %(w/v) $HA_{40}HD_{15}MA_{18}$ (a) or $HA_{80}DD_{15}MA_{13}$ (b) were 3D-printed into a filled flat cylinder, into which iMSC#3 cells ($3.75x10^{7}$ cells mL^{-1}) were injected in a heart shape in a second bioprinting step. (a, b) After 24 h the viability was analyzed via live-dead staining and images were taken with a confocal laser scanning microscope using a 10x objective. Living cells were stained with Calcein-AM (green), while dead cells can be seen in red (propidium iodide). All nuclei were stained with Hoechst 33342 (blue).

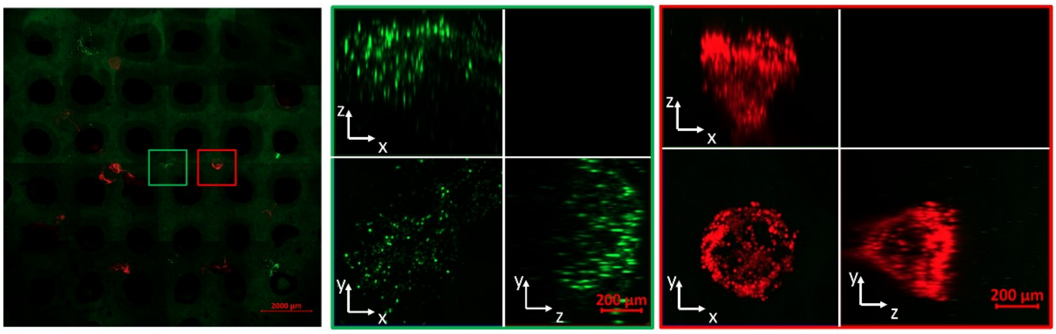

Figure S15. Confocal laser scanning microscopic image of an $HA_{80}DD_{15}MA_{13}$ hydrogel printed in a grid structure in a first step, with iMSC#3 cells labelled in red and KG-1a cells labelled in green injected into the nodes of the hydrogel grid in a second step (compare Figure 2(d)). Overview image on the left was acquired in *tiles and positions* mode with a 2.5x objective. The areas outlined in green and red are enlarged in the middle panel (green frame) and the right panel (red frame) outlined in their respective colors. The close-ups show the assemblies of injected cells (KG-1a cells in green, iMSC#3 cells in red) in top (x/y) and orthogonal views (y/z and z/x), captured as z-stack in combination with the *tiles and positions* mode using a 10x objective. KG-1a and iMSC#3 cells were printed at cell densities of $1x10^{7}$ cells mL^{-1}.

Table S1. List of used primers for qPCR experiments.

**Target gene**	**Abbreviation**	**Primer sequence**		**Product length**	**Detected transcripts**
Homebox A9	HOXA9	Forward	CCACGCTTGACACTCACACT	164 bp	NM_152739.4
		Reverse	GTTGGCTGCTGGGTTATTGG		
Spi-1 proto-oncogene	PU.1	Forward	CAGCTCTACCGCCACATGGA	92 bp	NM_003120.3, XM_054369738.1, XM_054369739.1, NM_001080547.2,
		Reverse	TAGGAGACCTGGTGGCCAAGA		
Collagen type I alpha 1 chain	COL1A1	Forward	GATTCCCTGGACCTAAAGGTGC	107 bp	XM_054315082.1, XM_054315081.1, XM_011524341.2, XM_005257058.5, NM_000088.4
		Reverse	AGCCTCTCCATCTTTGCCAGCA		
Alkaline phosphatase	ALPL	Forward	GCTGTAAGGACATCGCCTACCA	131 bp	NM_001369805.2, XM_017000903.2, XM_054335748.1, NM_001369803.2, NM_001127501.4, NM_001177520.3, NM_000478.6, NM_001369804.2
		Reverse	CCTGGCTTTCTCGTCACTCTCA		
Beta-2-microglobulin	B2M	Forward	TGCTGTCTCCATGTTTGATGTATCT	86 bp	NM_004048.4
		Reverse	TCTCTGCTCCCCACCTCTAAGT		

Table S2. Self-healing efficiency of hydrophobically modified HA-based hydrogels after deformation in cylic strain experiments calculated as the ratio of initial G' before deformation and G' after deformation cycle 1 to 5.

	**Self-healing efficiency [%]**					
**Cycle**	**$HA_{40}HD_{10}$**	**$HA_{40}HD_{15}$**	**$HA_{80}HD_{10}$**	**$HA_{80}DD_{25}$**	**$HA_{40}HD_{15}MA_{18}$**	**$HA_{80}DD_{15}MA_{13}$**
1.	99,9	99,4	85,8	84,6	81,8	92,3
2.	101,2	99,5	89,8	91,1	82,9	99,7
3.	102,4	101,2	89,1	87,7	84,4	98,4
4.	103,3	100,2	87,9	86,0	84,7	95,4
5.	102,9	99,7	86,1	84,7	83,0	92,3

Table S3. Estimated mesh sizes in nm of photo-crosslinked bioinks.

**$HA_{40}HD_{15}MA_{18}$**		**$HA_{80}DD_{15}MA_{13}$**		**Bone marrow**
Crosslinking time	Mesh size [nm]	Crosslinking time	Mesh size [nm]	Mesh size [nm]
2 min	12.3	2 min	12.1	10.2 – 12.9
5 min	10.3	5 min	9.7	
10 min	8.9	10 min	8.5	

	**Storage modulus [kPa]**	**Crosslinking kinetics**	**Crosslinking type**	**Cell viability**
**GelMA**	0.1-30 [1]	fast	temperature dependent gelatin gelation (physical) + photopolymerization (covalent) [1]	high [2]
**HA derivatives-GelMA**	higher than pure GelMA, up to 60 [3–6]	fast	depending on HA derivatives: mostly interpenetrating networks, physical crosslinking and photocrosslinking [5]	high [5,6]
**HA-Alginate**	< 1 [7] or 40-160 [8]	fast	ionic crosslinking, interpenetrating network [7,8]	high pre-printing [8] or intermediate [7]
**HAMA**	0.02-2.6 [9]	fast	physical crosslinking via high molecular weight HA + photocrosslinking [9]	intermediate [9]
**HA-Tyramide**	0.09-1.61 [10]	two step	step 1: enzymatic crosslinking, step 2: photocrosslinking [10,11]	high at optimized printing parameters and media [11]
**HA-Cysteine**	2.45-4.01 [12]	gelation time dependent on KI concentration	KI-catalyzed disulfide crosslinking [12]	intermediate to high (dependent on cell type) [12]
**Current study HA-HD-MA HA-DD-MA**	2.3-7.0 after covalent crosslinking	fast, crosslinking degree determined by irradiation duration	physical (H-bonds + hydrophobic interactions) + photocrosslinking	dependent on cell type, bioprinting process and culture period, high viabilities can be achieved
**Bone marrow**	2-4 at physiological temperature [13]	n.a.	n.a.	natural tissue

Table S4. Characteristics of different bioinks and comparison to bone marrow. n.a. = not applicable

References

[1] R.N. Ghosh, J. Thomas, V. B. R., D. N. G., A. Janardanan, P.K. Namboothiri, M. Peter, An insight into synthesis, properties and applications of gelatin methacryloyl hydrogel for 3D bioprinting, Mater. Adv. 4 (2023) 5496–5529. https://doi.org/10.1039/D3MA00715D.

[2] M.-R. Dobrisan, A. Lungu, M. Ionita, A review of the current state of the art in gelatin methacryloyl-based printing inks in bone tissue engineering, Virtual and Physical Prototyping 19 (2024). https://doi.org/10.1080/17452759.2024.2378003.

[3] Y. Wang, Y. Chen, J. Zheng, L. Liu, Q. Zhang, Three-Dimensional Printing Self-Healing Dynamic/Photocrosslinking Gelatin-Hyaluronic Acid Double-Network Hydrogel for Tissue Engineering, ACS omega 7 (2022) 12076–12088. https://doi.org/10.1021/acsomega.2c00335.

[4] D. Petta, U. D'Amora, L. Ambrosio, D.W. Grijpma, D. Eglin, M. D'Este, Hyaluronic acid as a bioink for extrusion-based 3D printing, Biofabrication 12 (2020) 32001. https://doi.org/10.1088/1758-5090/ab8752.

[5] K. Martyniak, A. Lokshina, M.A. Cruz, M. Karimzadeh, R. Kemp, T.J. Kean, Biomaterial composition and stiffness as decisive properties of 3D bioprinted constructs for type II collagen stimulation, Acta biomaterialia 152 (2022) 221–234. https://doi.org/10.1016/j.actbio.2022.08.058.

[6] S. Asim, T.A. Tabish, U. Liaqat, I.T. Ozbolat, M. Rizwan, Advances in Gelatin Bioinks to Optimize Bioprinted Cell Functions, Advanced healthcare materials 12 (2023) e2203148. https://doi.org/10.1002/adhm.202203148.

[7] C. Galocha-León, C. Antich, A. Voltes-Martínez, J.A. Marchal, M. Mallandrich, L. Halbaut, E.B. Souto, P. Gálvez-Martín, B. Clares-Naveros, Human mesenchymal stromal cells-laden crosslinked hyaluronic acid-alginate bioink for 3D bioprinting applications in tissue engineering, Drug delivery and translational research 15 (2025) 291–311. https://doi.org/10.1007/s13346-024-01596-9.

[8] I. Gorroñogoitia, S. Olza, A. Alonso-Varona, A.M. Zaldua, The Effect of Alginate/Hyaluronic Acid Proportion on Semi-Interpenetrating Hydrogel Properties for Articular Cartilage Tissue Engineering, Polymers 17 (2025). https://doi.org/10.3390/polym17040528.

[9] M.T. Poldervaart, B. Goversen, M. de Ruijter, A. Abbadessa, F.P.W. Melchels, F.C. Öner, W.J.A. Dhert, T. Vermonden, J. Alblas, 3D bioprinting of methacrylated hyaluronic acid (MeHA) hydrogel with intrinsic osteogenicity, PLOS ONE 12 (2017) e0177628. https://doi.org/10.1371/journal.pone.0177628.

[10] D. Petta, D.W. Grijpma, M. Alini, D. Eglin, M. D'Este, Three-Dimensional Printing of a Tyramine Hyaluronan Derivative with Double Gelation Mechanism for Independent Tuning of Shear Thinning and Postprinting Curing, ACS Biomater. Sci. Eng. 4 (2018) 3088–3098. https://doi.org/10.1021/acsbiomaterials.8b00416.

[11] D. Petta, A.R. Armiento, D. Grijpma, M. Alini, D. Eglin, M. D'Este, 3D bioprinting of a hyaluronan bioink through enzymatic-and visible light-crosslinking, Biofabrication 10 (2018) 44104. https://doi.org/10.1088/1758-5090/aadf58.

[12] S. Tavakoli, A. Kocatürkmen, O.P. Oommen, O.P. Varghese, Ultra-Fine 3D Bioprinting of Dynamic Hyaluronic Acid Hydrogel for in Vitro Modeling, Advanced materials 37 (2025) e2500315. https://doi.org/10.1002/adma.202500315.

[13] L.E. Jansen, N.P. Birch, J.D. Schiffman, A.J. Crosby, S.R. Peyton, Mechanics of intact bone

marrow, Journal of the mechanical behavior of biomedical materials 50 (2015) 299–307. https://doi.org/10.1016/j.jmbbm.2015.06.023.
